# Using Domain Based Latent Personal Analysis of B Cell Clone Diversity Patterns to Identify Novel Relationships Between the B Cell Clone Populations in Different Tissues

**DOI:** 10.3389/fimmu.2021.642673

**Published:** 2021-04-01

**Authors:** Uri Alon, Osnat Mokryn, Uri Hershberg

**Affiliations:** ^1^ Department of Human Biology, Faculty of Sciences, University of Haifa, Haifa, Israel; ^2^ Department of Information Systems, Faculty of Social Sciences, University of Haifa, Haifa, Israel

**Keywords:** Immunity, Complexity, B cell, diversity, Long tail distribution, Population comparison

## Abstract

The B cell population is highly diverse and very skewed. It is divided into clones (B cells with a common mother cell). It is thought that each clone represents an initial B cell receptor specificity. A few clones are very abundant, comprised of hundreds or thousands of B cells while the majority have only a few cells per clone. We suggest a novel method - domain-based latent personal analysis (LPA), a method for spectral exploration of entities in a domain, which can be used to find the spectral spread of sub repertoires within a person. LPA defines a domain-based spectral signature for each sub repertoire. LPA signatures consist of the elements, in our case - the clones, that most differentiate the sub repertoire from the person’s abundance of clones. They include both positive elements, which describe overabundant clones, and negative elements that describe missing clones. The signatures can also be used to compare the sub repertoires they represent to each other. Applying LPA to compare the repertoires found in different tissues, we reiterated previous findings that showed that gut and blood tissues have separate repertoires. We further identify a third branch of clonal patterns typical of the lymphatic organs (Spleen, MLN, and bone marrow) separated from the other two categories. We developed a python version of LPA analysis that can easily be applied to compare clonal distributions - https://github.com/ScanLab-ossi/LPA. It could also be easily adapted to study other skewed sequence populations used in the analysis of B cell receptor populations, for instance, k-mers and V gene usage. These analysis types should allow for inter and intra-repertoire comparisons of diversity, which could revolutionize the way we understand repertoire changes and diversity.

## Introduction

The extremely high diversity of B cell and T cell receptors in the immune repertoire is key for immune function and protection from disease (1). Clear links have been made between its loss with age and human frailty (2–6), and several tools have been put forth to assess overall levels of repertoire diversity and its changes (7, 8). Building on these, we suggest here a method to directly compare the distribution pattern of sequence and clonal abundance. The patterns of B cell and T cell diversity follow a long-tailed distribution (9, 10) resembling a Zipf distribution (11). Zipf-like probability distributions are found in many social systems (for example, cities’ sizes and the distribution of Twitter followers), cognitive systems (word usage), and biological systems (species abundance, gene expression, inter heartbeat intervals) (11–16). The long-tail distribution results from the auto-catalytic nature of the systems underlying these distributions, where abundance and proliferation are linked to use and functionality (17, 18). In all of these systems, things that are abundant in a given context become more abundant, and abundances can change rapidly when contexts change. For instance, in the T cell repertoire, it has been shown that the long-tail distribution of T cell receptors makes it equally likely for an antigen to be presented to rare or common T cell receptor types (1). In this way, the T cell distribution allows both rare and common T cells to be tested for their affinity when the immune system is activated and searching for a receptor response to disease.

When comparing these distributions that are long-tailed and far from normally distributed, we still tend to use tools that depend on normalcy and focus on the most abundant members of the distribution. Moreover, in most cases, it is hard to quantify a negative difference (i.e., when something is missing) when comparing distributions. We suggest a novel method, domain-based Latent Personal Analysis (LPA), recently developed to analyze texts (19), which considers both the head and the tail of the distribution and can compare both its abundancies and missing components.

LPA is a domain-based exploration method that creates for each entity in the domain two attributes. The first is the entity’s distance from the domain, encompassing how an entity is different from the domain. The second is the entity’s signature in the domain, consisting of the elements that make the entity different. LPA considers both the head and the tail of the domain’s elements distribution and can compare both its abundancies and missing components. Thus, an entity’s signature contains both domain-rare elements that are significantly more prevalent in the entity and domain-popular elements that are either absent or significantly less common in the entity than in the domain. Originally designed to analyze patterns of text and identify their authors, the hypothesis at the heart of LPA is that personal signatures in a domain can be derived by defining how a person’s vocabulary *differs* most from the domain’s vocabulary (i.e., the vocabulary across all texts). If one negatively assumes no personal differences, then a personal vocabulary could be considered a random sample from the domain vocabulary. This would yield a minimal information loss when measured by a relative entropy distance, e.g., a Kullback-Leibler Divergence (20). By contradiction, personal differences are the elements that contribute most to the distance measured between a personal vocabulary and the domain. A personal signature consists of elements distinguishing the author: overused or underused words and their relative sign (plus or minus).

**Figure 1:**
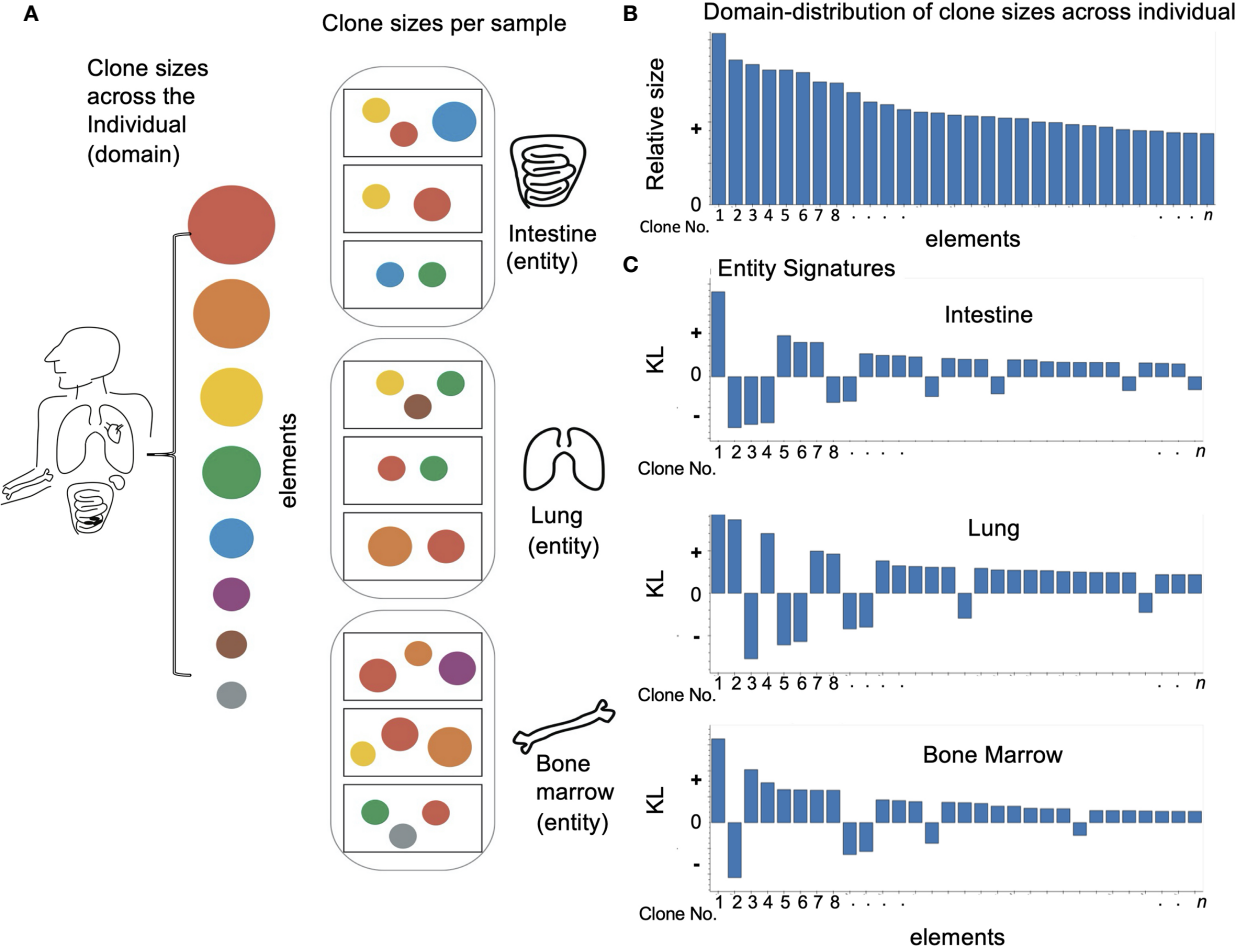
Applying domain based LPA to immune data: **(A)** Looking at an individual immune repertoire we can divide it into clones of different sizes (represented here by circles of different colors and sizes). When we look at different tissues (oval frames) we find they each have their own skewed set of clone types and clone sizes which differ from the overall picture and each other. The samples in each tissue (rectangular frames) show and even more skewed image of the overall repertoire. **(B)** To apply domain based LPA we must first define a domain – here the individual immune repertoire and the elements of which it is comprised – clones. **(C)** We can then create for each sub-category or entity in the domain a signature – here we see a signature for every tissue. As described in *Methods* the signature has two values - a size which reflects how different the element is in a given entity. i.e. how different clone size is in a given tissue and a sign: “+” if the clone is overrepresented compared to the overall repertoire and “–” if the clone is absent or underrepresented.

Similarly, we ask here if the distribution of clones (sets of B cells or T cells with the same mother cell and thus the same or related immune receptor) observed in a specific tissue match or differ from the overall distribution of clones observed in a person. Here, the *domain* is a *person*, that is, the overall distribution of clones observed in that person, the *elements* are the *clones*, and the *entities* in the domain, for which we compute the distance and signature features, are the *tissue samples*. In some cases, we consider a collection of samples that belong to the same *tissue* as an *entity* (**Figure 1**). That is, we group all the elements of the samples that were sampled from the same tissue and consider this newly aggregated collection as an entity. When tissues are considered entities, the domain is still the person’s distribution, but there are much fewer entities, the tissues. In either case, the domain is the collection of clones in a person, and the entities are either each tissue sample’s distribution of clones, if the entities are tissue samples, or the tissue’s distribution of clones, if the entities are the tissues. An LPA’s entity signature improves our understanding of the clones that compose it in two ways. First, as the signature is a trait of the entity (i.e., the tissue or tissue sample) relative to the person, it allows us to compare tissues and tissue samples to each other and to the overall B cell repertoire. Second, it highlights how the entity is different from the collection of all sampled clones from the person, which we term the domain, or how the entity is different from other entities. We can analyze each tissue sample or tissue signature and identify which clones are prevalent and which are lacking compared to other tissues or samples, and hence in the person. The reason is that if a certain clone is very dominant in the person but absent from the tissue, that tells as much about the tissue as if a specific clone is infrequent in the individual but very abundant in the tissue. In this way, the signature gives weight to both the highly abundant head of the individual’s distribution clones and its long tail. This also holds for when the entities considered are the tissue samples, in which case LPA creates a spectral mapping of the samples with regards to the individual’s clonal distribution.

In the research below, we show how applying LPA to the B cell populations of six individuals scanned across eight different tissues allows us to observe both known relationships between repertoires found in gut and blood tissues (21) and new relationships between the different lymphoid tissues, as observed by the differences in clonal distribution and clonal dominance. Beyond these specific results, we further show how these methods could be applied to other comparisons, with our novel python code here - https://github.com/ScanLab-ossi/LPA, and how to identify cases when under-sampling of populations makes them untenable in the immune and other domains.

## Methods

### Comparing the Repertoires Found in the Different Tissues of an Individual

The analyses performed here were done on the high throughput sequenced B cell receptor populations from eight tissues of six individuals at different sampling levels [described in Meng et al., 2017 (21) see - **Supplemental Table 1**]. For each individual, we created a table of all the individual clones annotated for their size in unique sequence instances or copies per sample and across tissues and the individual (**Supplemental Materials File 1** **- clone size source data**). The size of the clone in unique instances is the number of unique sequences a clone has per sample (21).

### Applying LPA to Immunological Data

To apply LPA (19) to immune cell populations, we looked at the abundance of all clones in a person across tissues and samples. Clone abundance was defined as the number of unique sequences in a sample, aggregated over all samples [i.e., the number of instances of a clone in a person (21)]. Counting each clone as an element, we treated each person as a single domain and explored this domain of clones as they appear in samples or in tissues. We computed the domain distance and signatures for entities by considering each tissue as an entity or, as proved more fruitful, by considering each tissue sample as an entity. Following the results in Mokryn and Ben-Shoshan (19), We converted each entity *via* LPA to a vector that describes the entity in relation to the domain. LPA then finds the distance of each entity from the domain. An entity’s distance feature is a value between 0 and 1 that is a measure of how similar the entity is to the domain (i.e., in our study, how similar is the snapshot of clone abundances in a tissue sample to that found in the individual). The distance is calculated as the sum of the distances between the relative weights of the same elements (clones) in the two vectors – the domain and the entity. Clones that are prominent in the entity but not in the person, and clones that are prominent in the person but not in the entity, contribute to the entity’s distance. For distance calculation, LPA uses KLDϵ, a variant of Kullback-Leibler Divergence with a back-off model, defined by Bigi (20). Specifically, the Kullback-Leibler Divergence (22) measures the distance between two distributions of the same length. Yet, in LPA, two vectors are not necessarily of the same length, as not all the clones may appear in every sample or every tissue. To account for the large variance in vector length, KLDϵ pads the vectors with a constant value ε instead of zeros. This, however, also means that the extended vector, *V_e_*, is no longer a probability vector - the sum of all coordinates is now larger than 1. We correct this by multiplying all non-ε frequencies by a normalization coefficient β. This normalization coefficient is given by the formula β = 1−N_ε_, where N is the number of missing elements in each vector.

To decide on the adequate value of ε, we required ε to be less than the frequency of a single clone in the entire individual and still large enough so that KLD gives results in the range [0, 1]. We, therefore, chose 1/(number of unique clones in the individual* 2) (19). We define an entity’s signature as the top K clones that contributed most to the entity’s distance from the domain. Thus, an entity’s signature defines how an entity is *different* from the person. To determine signature length K, we considered the great variance in clones and differences in sampling levels. Each individual had different numbers of clones and different levels of sampling. Also, single samples could never truly sample the full diversity of a person. Signatures that are too short would cause all samples to be extremely different, simply because of the abundance of unique clones. Signatures that are too long would overemphasize noise. Therefore, we determined a specific signatures’ length for each individual, based on the threshold number of elements that describe 0.5 of the distance between the individual, that is, the domain, and its entities (i.e. tissue samples) (19). After this point, specific elements hardly add to each tissue sample’s distance from the individual and are not sample-distinguishing elements. Hence, they are not part of the tissue sample’s signature (see **Supplemental Materials File 2** **- Signature cutoff Tables** and **Supplemental Materials File 3** **- Signature cutoff interactive Figures**).

The signature, consisting of the K clone-elements that most differentiate the tissue sample or tissue from the individual, is a vector of tuples. Each tuple in the signature consists of an element name (a unique clone ID in this case), its distance from the domain (the difference between local abundance and global abundance), and a sign, indicating whether, in the entity, it is a locally missing clone or a locally over-abundant clone. To determine the similarity between entities and see how different entities are from each other, we calculate the distance between the entities’ signatures. Recall that the signatures are vectors of tuples: <element, element-KLD> with the element-KLD denoting the KLD distance between the element’s global domain weight and its local-entity weight. The element-KLD is a value in the range [-1,1] and has a negative value when the element is locally missing or rare. Clearly, when two signatures have the same elements with opposite element-KLD signs, i.e., in one entity, the element is more frequent than in the domain, and in the other, it is absent, their relative distance needs to increase. To that end, positive element-KLD values are incremented by one, while negative element-KLD values are decremented by minus one. These changed signatures are termed the sign-corrected signatures. The similarity is then determined by calculating the L_1_-norm distance between the sign-corrected signatures. Having calculated for each tissue sample, and set of tissue samples in a single tissue, their sign corrected signature distance from the individual’s clone frequency distribution, we next calculated the L1 distances of every signature-pair to compare the tissue samples and tissues directly.

### The Python Code

LPA computes KLD distances between vectors on a per-element basis, performing simple arithmetic operations and logarithms. The computation is therefore the order of the number of elements in the domain, n. The signatures are of size K<<n. Given there are q entities, calculating the distance between all entities requires O(K*q^2). Hence, as detailed in (19), LPA is fast and has very low memory requirements. In order to more easily apply the algorithm to immunological data, we wrote a Python implementation of LPA (19). The python implementation assumes a correct format (element id, category id, and frequency in category columns) and automates all steps of the calculation. The python implementation is completely agnostic to the input data, as long as it corresponds with the proper format. That means one simply needs to define a domain with various entities and elements that appear in varying frequencies and input the data in the proper format to receive results. The python implementation allows for quick and simple results in many forms: A summary of every entity’s distance from the world, the signature of every entity in the domain, or the distance between every entity-pair. Furthermore, one can manipulate the length of the signature, the normalization factor, and any part of the code they see fit. The code is open source can be found in https://github.com/ScanLab-ossi/LPA.

## Results

### The Gut and Blood Compartments Are Separate

**Figure 2:**
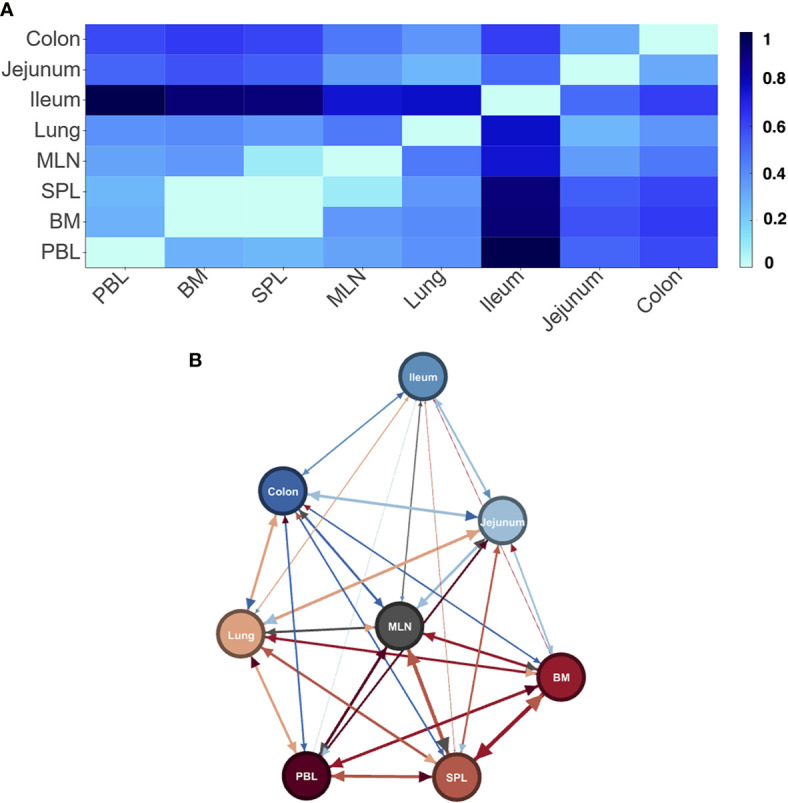
Differences in LPA signatures of tissue based entities capture tissue differences in D207. Heatmap **(A)** and network representation **(B)** of the signature pair distances of tissue entities of donor D207. Darker colors (heat map) and longer/wider edges (network) signify greater difference between signatures.

As a first step, we wished to see if our new method could identify tissues from samples and differentiated between the tissues in the gut and the other “blood” tissues as we had observed before (21). Combining all samples to consider each tissue as a separate entity, we found a clear signature for each tissue (**Supplemental Materials File 4** **- by tissue entity distance tables**). As expected in D207, which was sampled sufficiently to observe all expanded clones (21), we found that indeed the gut tissues (ileum, jejunum, and colon) were the most distant from the blood tissues (**Figure 2**). We could also observe two new relationships (1) that the lymphatic tissues (BM, SPL, MLN) were most close to each other, and (2) all tissues were quite distant from each other and the lymphatic tissues. In the other individuals, although the tissues are clearly delineated, we find less clear patterns (**Supplemental Materials File 5** **- by tissue distance heatmaps and networks**). This is not surprising given that none of the other individuals were sufficiently sampled to describe even the expanded clone diversity in their B cell receptor populations (21) and only d181, which is relatively well sampled, showed a progression of its signature threshold (see *Methods*) that was similar to that in D207 (**Supplemental Materials File 3** **- Signature cutoff interactive Figures**).

**Figure 3:**
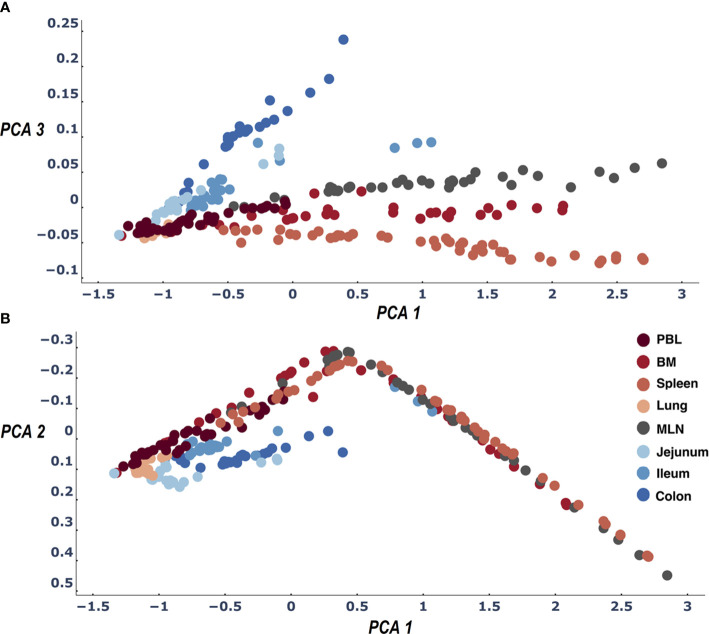
Three dimensions of the PCA of the LPA signature differences across all sample pairs: PCA1 and PCA2 **(B)** and PCA1 and PCA3 **(A)**. The first three dimensions of the PCA account for>95% of the variance. When we color samples by their tissue a clear pattern emerges differentiating between all tissues. We find the expected difference between gut and blood tissues (21) and in addition observe that the samples of the lymphatic tissues (Spleen, MLN, BM) are differentiated by their clone size LPA signatures.

To identify the differences between tissues more clearly, we drilled down into our data and treated each sampling experiment as an entity. The tissue categories are in some sense arbitrary/circular as they depend on our nomenclature. The only real individual entity is the experimental sample. On the other hand, each sample is clearly under-sampling the clone diversity of the repertoire as a whole. We therefore wanted to test to what extent the method would identify and differentiate individual tissues when we do not combine all the samples into a single tissue entity but rather the samples are the entities. We created a distance matrix table of all samples (**Supplemental Materials File 6** **- by sample entity distance tables**). A PCA of these distances accounted for more than 95% of the variance in its first 3 dimensions. We now see much more clearly for both D207 and D181 that the blood tissues diverge from the gut tissues. In addition, we find that the central lymphatic tissues (BM, Spleen and MLN) diverge from the lung and blood, each of which have their own cluster in the PCA (**Figure 3** and **Supplemental Materials File 7** **- figures of 2D PCA1_PCA2_PCA3 comparison based on sample distances** and **Supplemental Materials file 8** **- 3d interactive figures of PCA based on sample distances**). Interestingly if the test signature is calibrated correctly (see *Methods*) even the under sampled individuals (D145,D149, D168,D182) show similar if less precise patterns of gut tissues and lymphatic tissues (see **Supplemental Materials File 8** **- 3d interactive figures of PCA based on sample distances)**.

### Under Sampling Gives Illusion of Complete Description of Repertoire but Overly Long Signature Choice Only Degrades Signal

Although we can see some indication of the patterns described above, D145,D149, D168 and D182 (21) appear to be insufficiently experimentally sampled to clearly show the patterns of clonal diversity across tissues observed above in D207 and D181. When thresholding the signature length as in (19) (see *Methods*) the PCA of the between sample differences in D207 and D181 the 1^st^ 3 dimensions of the PCA account for ~ 95% of the variance in the differences. At the same time in the other, less sampled, individuals the variance accounted for is between 38% and 64% (see **Supplemental Table 2** **- signature/variance**). An additional phenomenon that could indicate the lack of sampling is that we very rapidly reach a plateau of similarities in the under-sampled individuals. The domain is more easily described by the few entities from which it is made. It is tempting then to lengthen the signature to the size of the entire repertoire as it seems feasible to do so. However, we find that this additional information has harmful effects. It is true that when we lengthen the signature in D145, D149, D168, and D182 the first three dimensions of the calculated PCAs account for a greater portion of the variance (about 87% - see **Supplemental Table 2**). However, when we look at the shape of those PCAs we find that all the tissues are bunched together and the PCAs in general have less structure than those from signatures with the 0.5 cutoff (see **Supplemental Materials File 9** **- long signature interactive 3d PCA**). We therefore conclude that by lengthening the signatures beyond the 0.5 cutoff (see *Methods*) added very little new information and instead added noise to our analysis of the under sampled repertoires.

### The Lymphatics Map the Person

**Figure 4:**
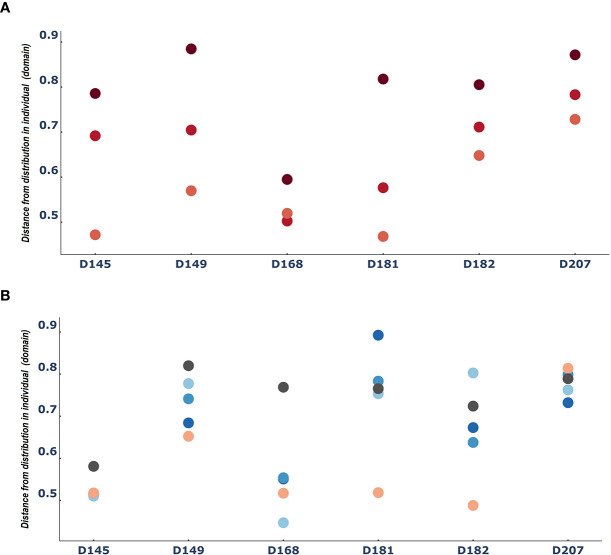
The spleen is reliably more like the entire repertoire than the blood: Median difference between tissue sample entities and the entire domain [i.e. the distribution of clones in a person) **(A)** for PBL(blood), spleen and BM (bone marrow) and **(B)** for the rest of the tissues]. Dots are colored by tissue type as in **Figure 3**.

Another way to consider differences between entities is to ask how different each entity is from the domain. In this case, we measured how different each tissue sample was from the individual. When we did this, we found again an indication of the consistent role of the lymphatics. Across all six individuals we found that the spleen samples were the most similar to the individual, followed by the bone marrow samples. In contrast, the blood samples were the most distant from the individual (**Figure 4A**). The patterns of distances of each tissue (lung and the 3 gut tissues) from the overall repertoire was more diverse and changed from person to person (**Figure 4B**).

### No Single Tissue Dominates the Entire Individual

**Figure 5:**
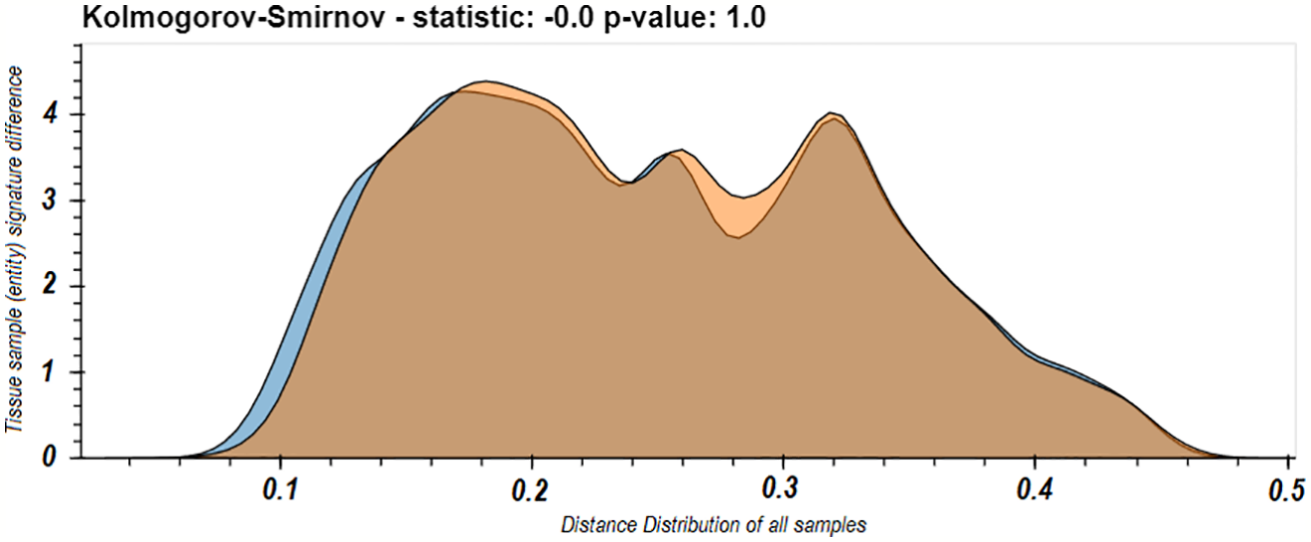
Distribution of all between sample entity signature differences in the immune domain for D207 with or without the spleen samples. The distribution of between sample signature differences comparing all samples in D207 (blue) and when all the samples in spleen are removed from the calculation of the immune domain (orange). In both cases the distances from samples in the spleen are not included.

These last results led us to worry that maybe the spleen dominates our view of the repertoire. One of the phenomena observed in the use of LPA is that if a domain is dominated by a specific entity or set of entities, it can affect the resulted signatures. A dominating entity’s removal changes the domain’s elements distribution, and hence other entities’ distance from the domain and hence their signatures. To test that this is not the case in the immune repertoire and that removing any one tissue does not radically change the observed nature of the individual B cell clone domain we calculated the distance distributions of the signatures of the samples from each tissue in D207 to all the other samples in D207 with and without the spleen. We chose the spleen because it was the most sampled tissue and as we showed above (**Figure 3**), its samples’ signatures are closest to the individual’s domain of clones, and hence the spleen can potentially be a dominating entity. We found very clearly that there is no significant difference in the distribution of distances with and without the spleen (p>>0.05 following a Kolmogorov–Smirnov test). In fact, they appear to overlap nearly perfectly (**Figure 5**).

## Discussion

We have presented a novel method to compare the distribution of B cell receptor repertoires in a way that highlights their similarities and differences. LPA allows us to identify and differentiate populations and sub-populations of B cells with a specific clonal and clonal distribution imprint. This gives us several advantages over existing methods for describing similarity in clonal populations (21) and diversity (7, 8). First we can consider the nature of the whole distribution and identify specific elements or clones that influence the differences. Second we can see if these differences are the result of a rise or a lack in abundance. Finally, we can compare similarities in clonal patterns across tissues not just by one to one comparisons but overall for the whole immune repertoire. With LPA we were able to expand our understanding of the relationship between clonal populations in different tissues. We re-iterated previous observations regarding the separation of gut and blood repertoires and could also further pinpoint multi-tissue relationships. We found that there is a clear sub-cohort of lymphatic organs within the blood tissues and blood is separated from all organs (**Figure 3** and **Supplemental Materials File 8** **- 3d interactive Figures of PCA based on sample distances**). Within the lymphatics, the spleen clearly encompasses the closest picture of the entire repertoire (**Figure 4**). One that reflects the repertoire as a whole and does not skew the clonal distributions of the other tissues (**Figure 5**).

In terms of its effectiveness of use with existing means of experimental B cell receptor sequence sampling, we showed that LPA works even if each specific sampling experiment under-samples the diversity of the domain. What is important is that sampling overall (i.e., the number of samples) is sufficient. Lack of sampling can clearly be seen by: (1) speedy plateauing of % distance (**Supplemental Materials File 3** **- Signature cutoff interactive Figures)** and (2) low variance explained when performing a PCA off of the signature differences (**Supplemental Table 2**). Hearteningly, it appears that even when sampling is borderline or lacking, if signatures’ lengths (see *Methods*) are set correctly, some differences can still be observed. In general, and especially in such cases, it is very important to set a correct signature length as otherwise we found that noise is added to our comparisons and the clarity of our results degrades.

Our results suggest that LPA could be a very good method of comparing B cell repertoires. We have used here clone size distributions as our example element. However, B cell repertoires are skewed not only in clone size. For this reason, we are now working on applying LPA to comparing clonal levels of V gene usage and of k-mer sub-sequences of B cell repertoires, within individuals and potentially even between them. Unlike B cell clones these more complex (and sequence-specific) distributions are mostly shared between individuals and could potentially allow us to then create both inter and intra personal comparisons of repertoires, and also to ask questions about motifs and their abundance and effects on population size. To aid with the analysis of different kinds of B cell receptor sequence and B cell clone distributions we have created a python-based version of LPA here: https://github.com/ScanLab-ossi/LPA which accepts any tables of annotated distributions to create domains and sets or related entities for comparison.

## Data Availability Statement

The original contributions presented in the study are included in the article/**Supplementary Material**. Further inquiries can be directed to the corresponding author.

## Author Contributions

UA ran the analysis and created the python version of LPA. OM designed LPA and its testing methods. UH designed the analysis and comparison of B cell populations and modified LPA to be used with immune cell repertoires. UA, OM and UH wrote the manuscript. All authors contributed to the article and approved the submitted version.

## Funding

This research was funded by NIH P01 AI106697 and the European Union’s Horizon 2020 research and innovation program, under grant agreement No 825821.

## Conflict of Interest

The authors declare that the research was conducted in the absence of any commercial or financial relationships that could be construed as a potential conflict of interest.

## Supplementary Material

Supplementary Materials File 1 - clone size source data: The raw data from which the analysis was performed. Six tables (based on sequence data re-aligned with local alignment, one table for each donor from (21)). Columns show unique clone id and sample id in which the clone was found (the same clone can be found in multiple samples. Each clone’s size is counted two ways – by number of unique sequences (instance) and by total copies (copies). Finally the tissue in which the sample is found in mentioned.

Supplementary Materials File 2 -Signature cutoff tables: The fraction of the entity’s (tissue or tissue sample) signature distance from the individual domain at each signature length. Two tables for each donor from (21).

Supplementary Materials File 3 - Signature cutoff figures: Twelve interactive figures of **Supplementary Materials 2.**

Supplementary Materials File 4 - by tissue entity distance tables: Distances between the signatures of every two tissues. one table for each donor from (21). Csize – number of unique clones summed between every two tissues (i.e. max csize=signature size*2); distance – distance between tissues and the names of both entities (tissues) compared.

Supplementary Materials File 5 - by tissue distance heatmaps and networks: Heatmaps and Network representations as in **Figure 1** one set of figure for each donor from (21).

Supplementary Materials File 6 - by sample entity distance tables: Distances between the signatures of every two tissue samples. one table for each donor from (21). Csize – number of unique clones summed between every two tissues (i.e. max csize=signature size*2); distance – distance between samples followed by the sample ids and tissue source of both samples compared.

Supplementary Materials File 7 - figures of 2D PCA1_PCA2_PCA3 comparison based on sample distances: Relationships of the first three dimensions of the PCA for tissue sample signature differences calculated across each individual and color coded by tissue as in **Figure 3**. One set of figures for each donor from (21).

Supplementary Materials File 8 - 3D interactive figures of PCA based on sample distances: 3D interactive web based figures (.html) showing a movable 3drepresentation of the first three dimensions of the PCA for tissue sample signature differences calculated across each individual and color coded by tissue as in **Figure 3**. One figure for each donor from (21).

Supplementary Materials File 9 - long signature interactive 3D PCA: 3D interactive web based figures (.html) showing a movable 3drepresentation first three dimensions of the PCA for sample signature differences calculated across each individual and color coded by tissue as in **Figure 3**. Signatures in this analysis were calculated without the threshold for signature size described in *Methods*. One figure for each of the under-sampled donors from (21).
