# Supplementary material for: Using Domain Based Latent Personal Analysis of B Cell Clone Diversity Patterns to Identify Novel Relationships Between the B Cell Clone Populations in Different Tissues
Source: Front Immunol. 2021 Apr 1;12:642673. doi: 10.3389/fimmu.2021.642673 (PMC8047331; doi:10.3389/fimmu.2021.642673)
Supplement: Supplementary Materials File 3 — - Signature cutoff figures: Twelve interactive figures of Supplementary Materials 2. [file DataSheet_3.zip › Supplemental Materials file 3 - signature cutoff interactive figures/D145bytiss_sig_cutoff.html]

D145bytiss\_sig\_cutoff
