# Supplementary figures and images for: Using Domain Based Latent Personal Analysis of B Cell Clone Diversity Patterns to Identify Novel Relationships Between the B Cell Clone Populations in Different Tissues
Source: Front Immunol. 2021 Apr 1;12:642673. doi: 10.3389/fimmu.2021.642673 (PMC8047331; doi:10.3389/fimmu.2021.642673)

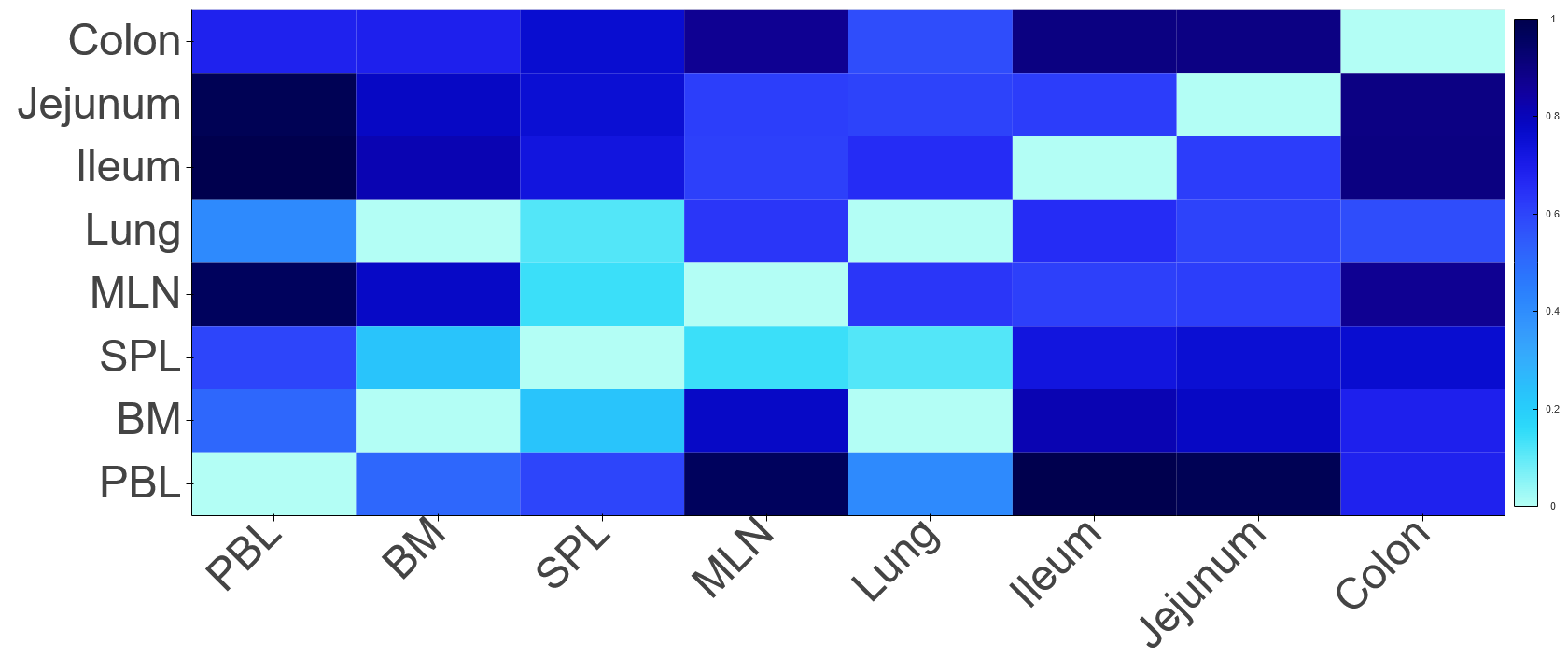

Supplement: Supplementary Materials File 5 — - by tissue distance heatmaps and networks: Heatmaps and Network representations as in Figure 1 one set of figure for each donor from (21). [file DataSheet_5.zip › Supplemental Materials file 5 -tissue distance heatmaps and networks/D181_bytiss_heatmap_HD.png]

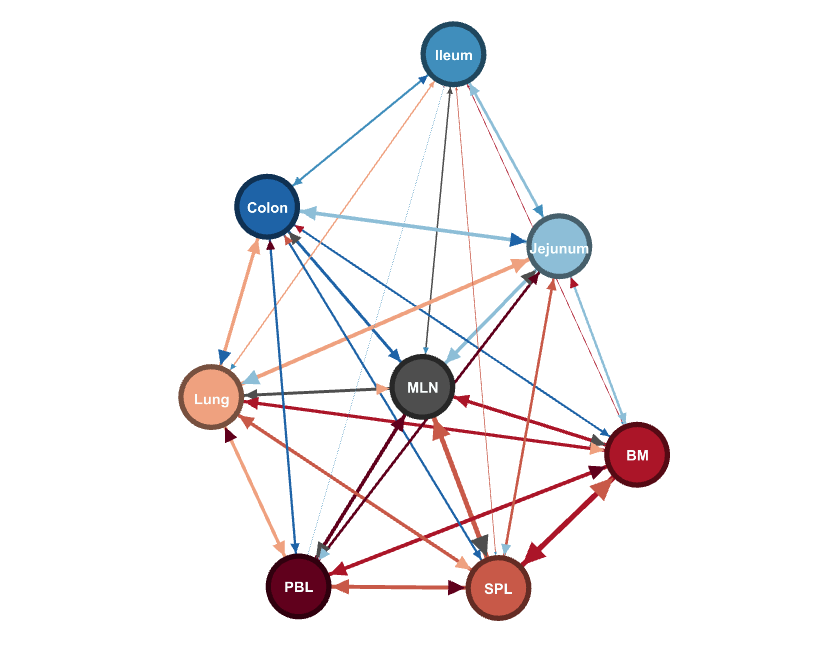

Supplement: Supplementary Materials File 5 — - by tissue distance heatmaps and networks: Heatmaps and Network representations as in Figure 1 one set of figure for each donor from (21). [file DataSheet_5.zip › Supplemental Materials file 5 -tissue distance heatmaps and networks/D207 by tissue graph.png]

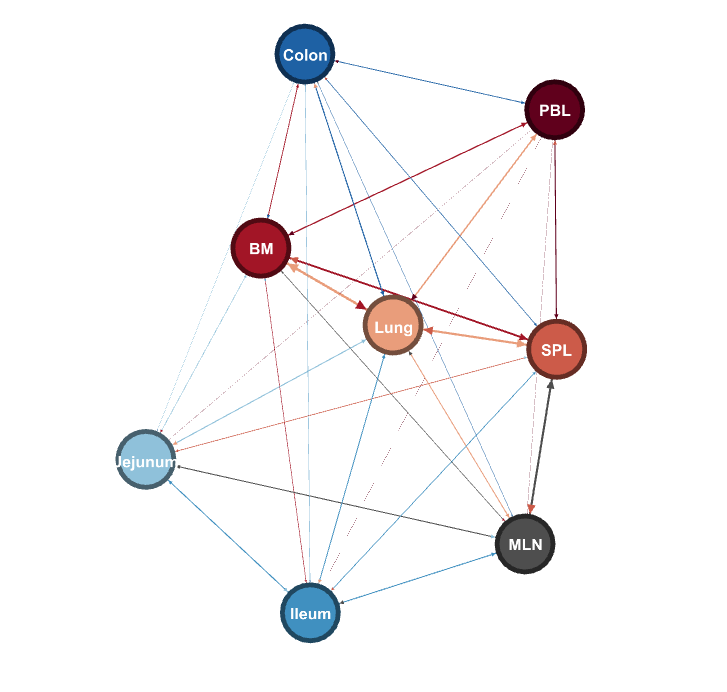

Supplement: Supplementary Materials File 5 — - by tissue distance heatmaps and networks: Heatmaps and Network representations as in Figure 1 one set of figure for each donor from (21). [file DataSheet_5.zip › Supplemental Materials file 5 -tissue distance heatmaps and networks/D181 by tissue graph.png]

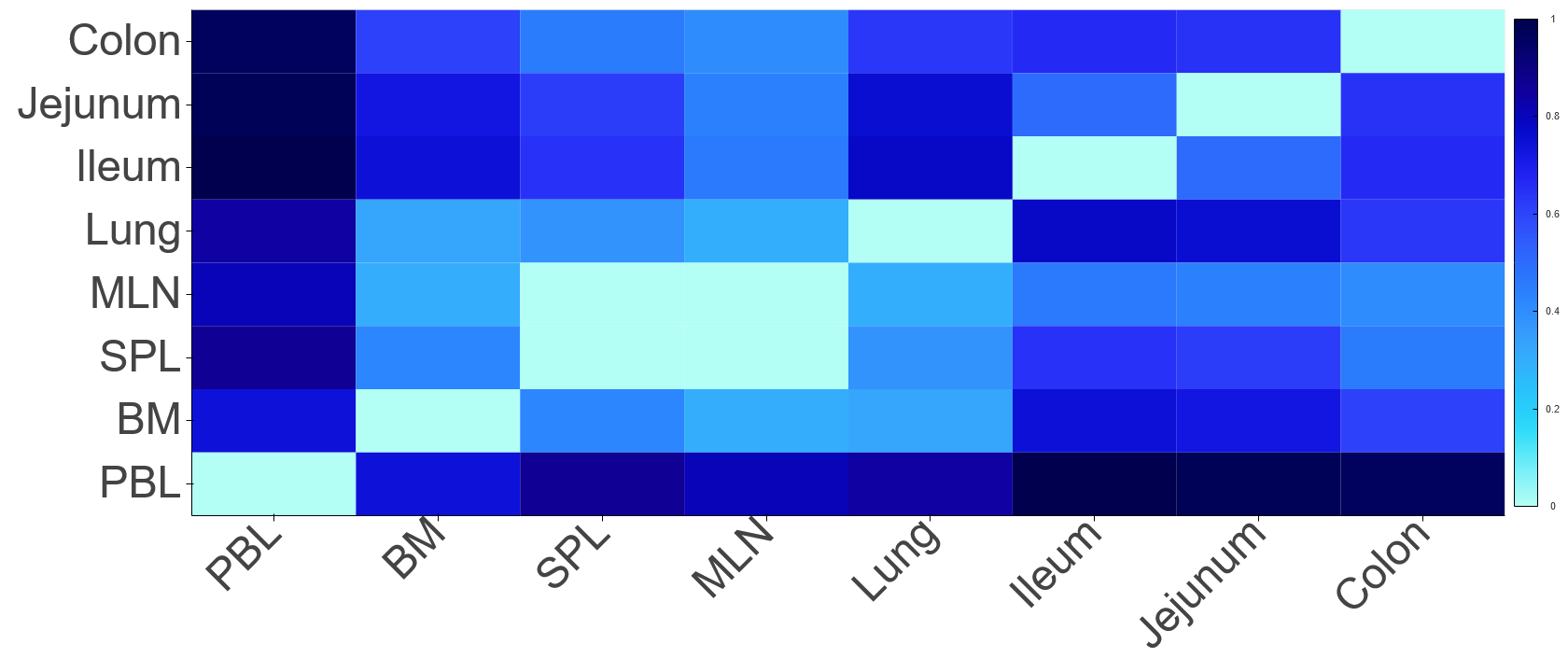

Supplement: Supplementary Materials File 5 — - by tissue distance heatmaps and networks: Heatmaps and Network representations as in Figure 1 one set of figure for each donor from (21). [file DataSheet_5.zip › Supplemental Materials file 5 -tissue distance heatmaps and networks/D145_bytiss_heatmap_HD.png]

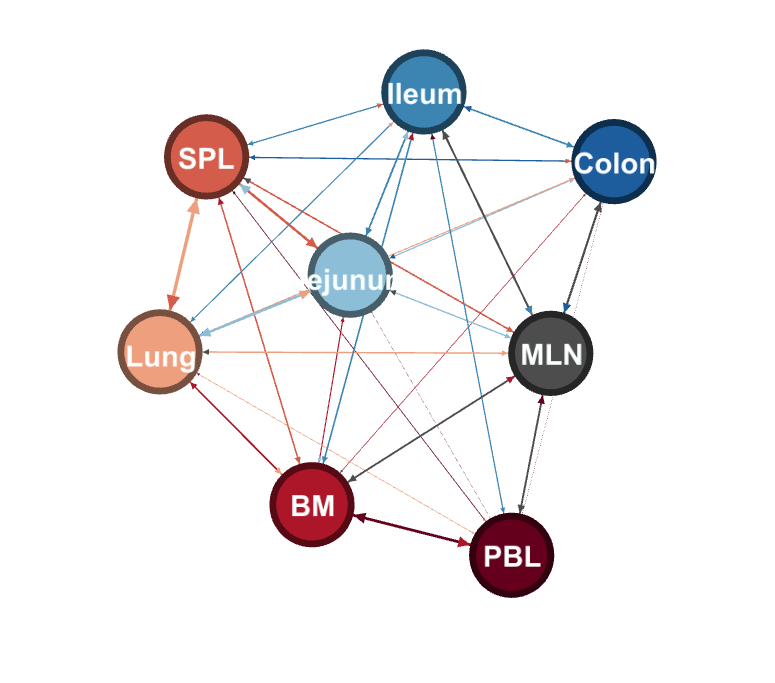

Supplement: Supplementary Materials File 5 — - by tissue distance heatmaps and networks: Heatmaps and Network representations as in Figure 1 one set of figure for each donor from (21). [file DataSheet_5.zip › Supplemental Materials file 5 -tissue distance heatmaps and networks/D168 by tissue graph.png]

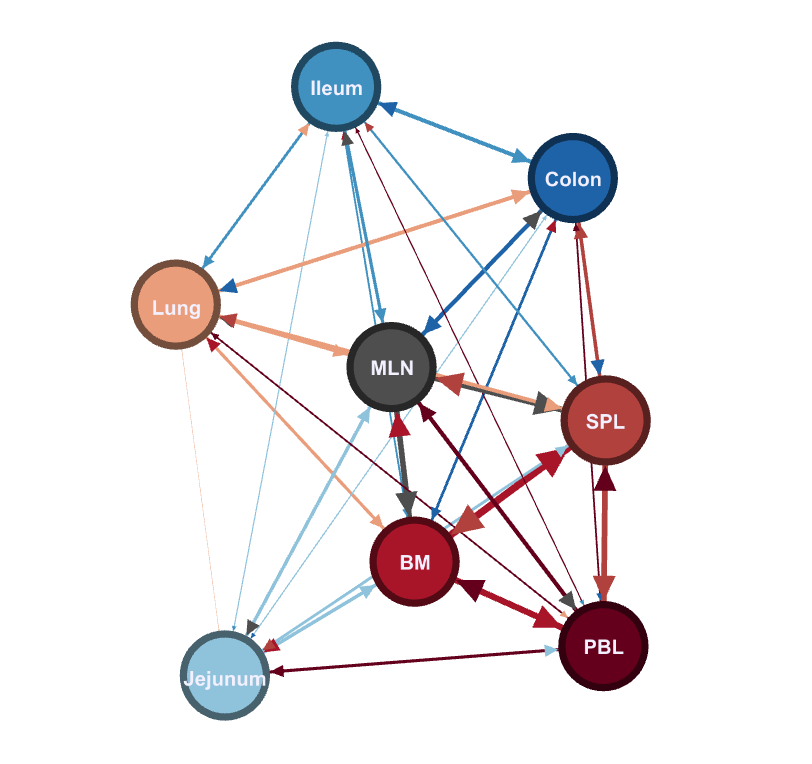

Supplement: Supplementary Materials File 5 — - by tissue distance heatmaps and networks: Heatmaps and Network representations as in Figure 1 one set of figure for each donor from (21). [file DataSheet_5.zip › Supplemental Materials file 5 -tissue distance heatmaps and networks/D182 by tissue graph.png]

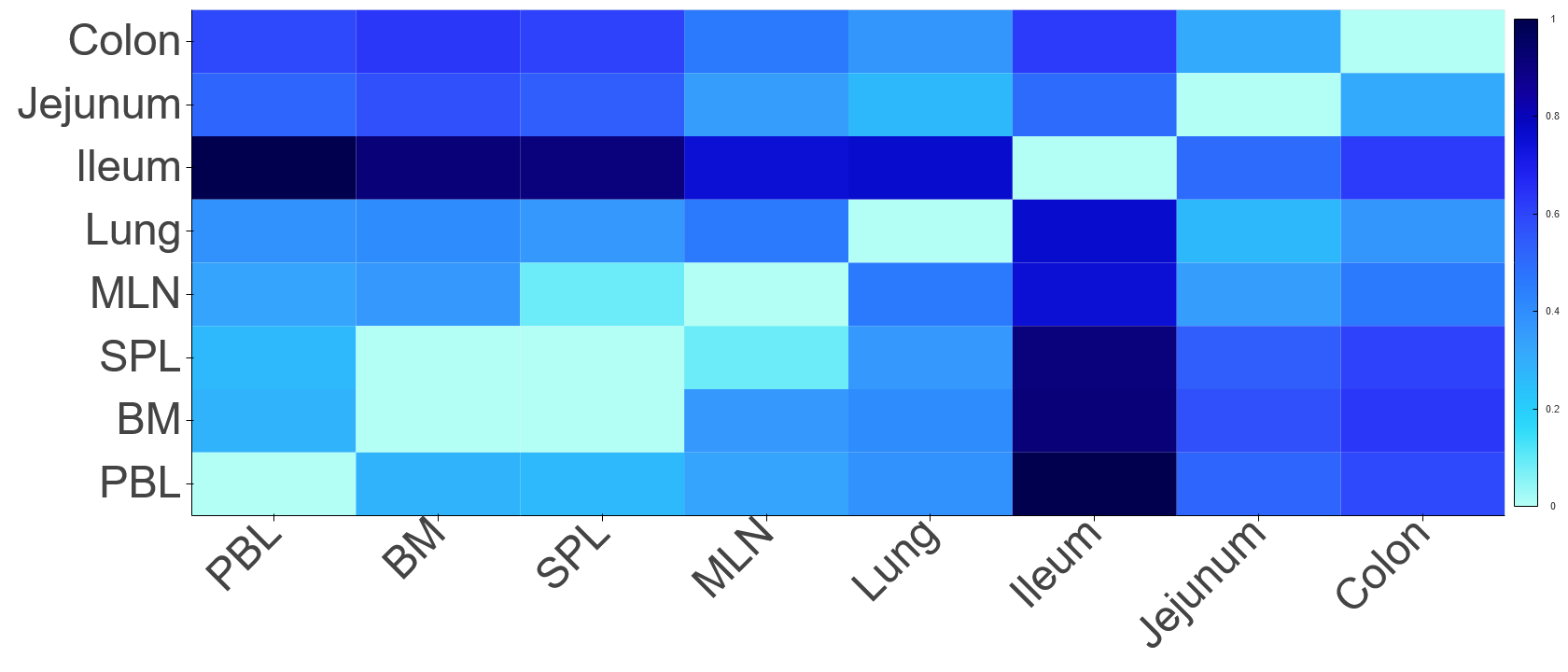

Supplement: Supplementary Materials File 5 — - by tissue distance heatmaps and networks: Heatmaps and Network representations as in Figure 1 one set of figure for each donor from (21). [file DataSheet_5.zip › Supplemental Materials file 5 -tissue distance heatmaps and networks/D207_bytiss_heatmap_HD.png]

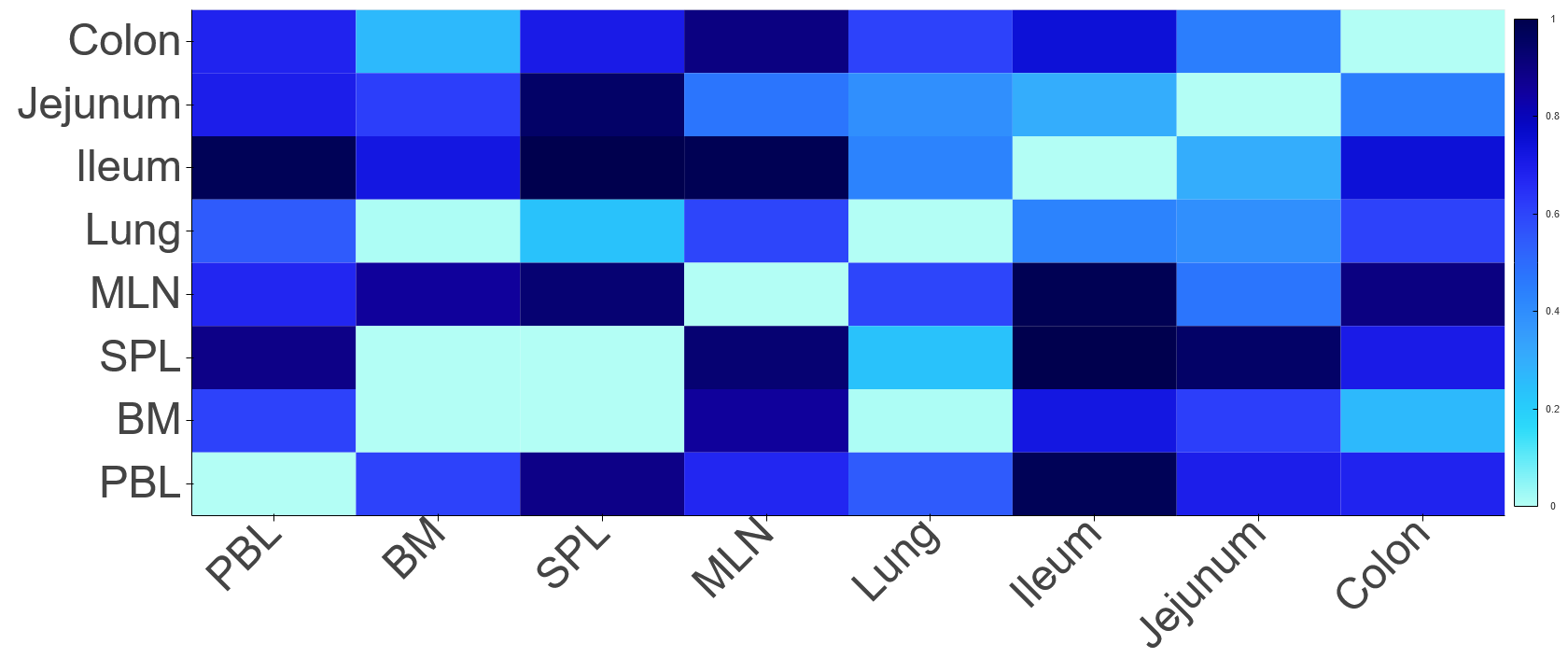

Supplement: Supplementary Materials File 5 — - by tissue distance heatmaps and networks: Heatmaps and Network representations as in Figure 1 one set of figure for each donor from (21). [file DataSheet_5.zip › Supplemental Materials file 5 -tissue distance heatmaps and networks/D149_bytiss_heatmap_HD.png]

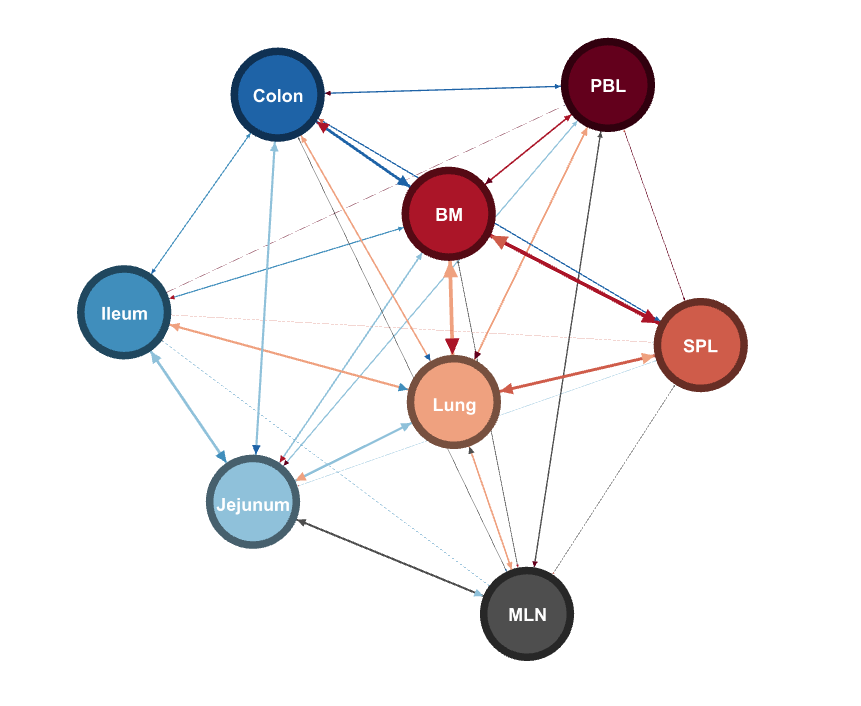

Supplement: Supplementary Materials File 5 — - by tissue distance heatmaps and networks: Heatmaps and Network representations as in Figure 1 one set of figure for each donor from (21). [file DataSheet_5.zip › Supplemental Materials file 5 -tissue distance heatmaps and networks/D149 by tissue graph.png]

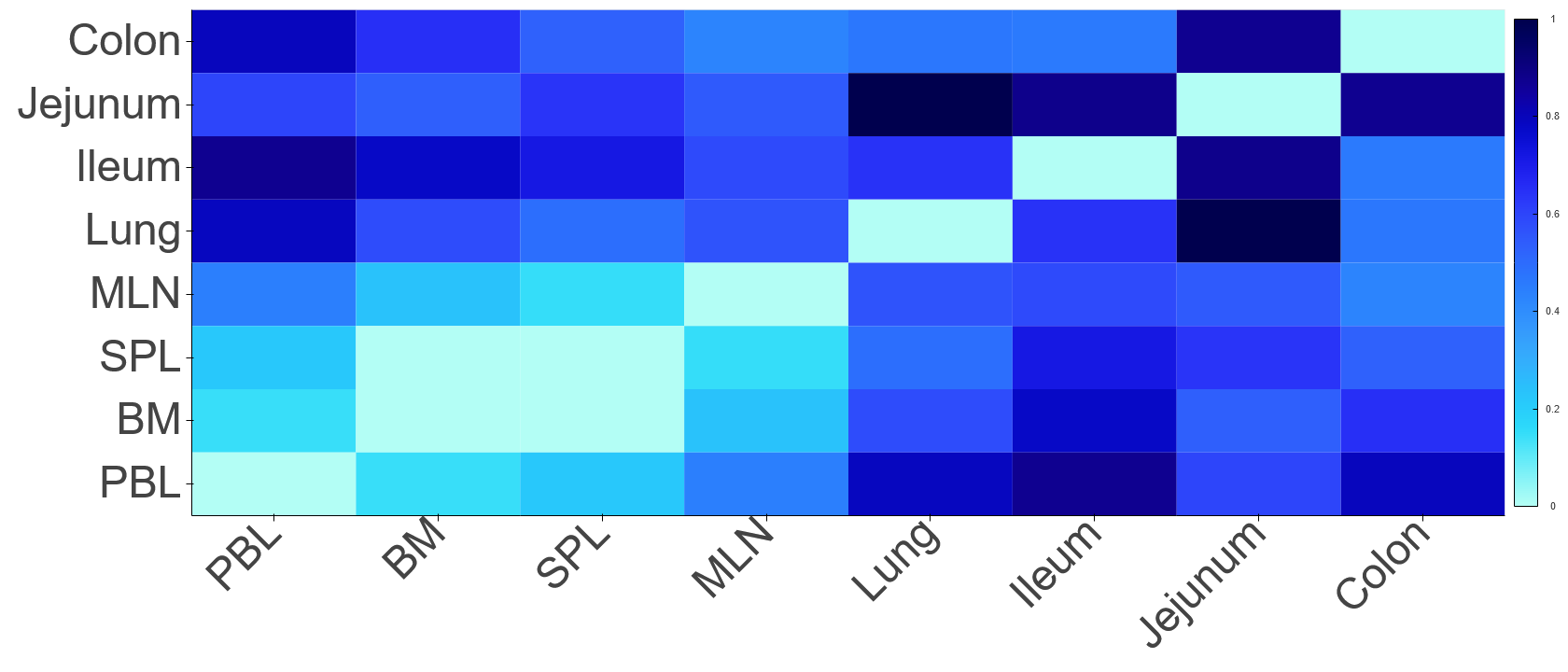

Supplement: Supplementary Materials File 5 — - by tissue distance heatmaps and networks: Heatmaps and Network representations as in Figure 1 one set of figure for each donor from (21). [file DataSheet_5.zip › Supplemental Materials file 5 -tissue distance heatmaps and networks/D182_bytiss_heatmap_HD.png]

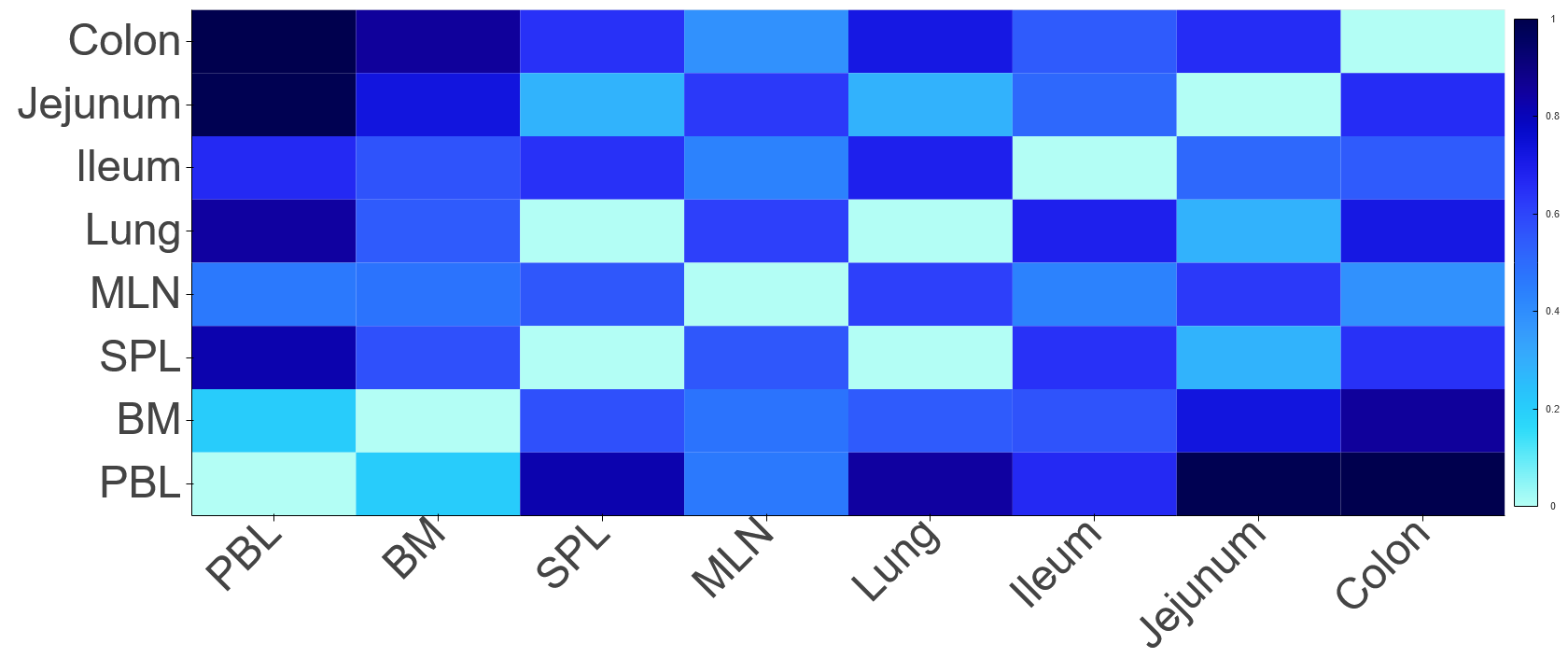

Supplement: Supplementary Materials File 5 — - by tissue distance heatmaps and networks: Heatmaps and Network representations as in Figure 1 one set of figure for each donor from (21). [file DataSheet_5.zip › Supplemental Materials file 5 -tissue distance heatmaps and networks/D168_bytiss_heatmap_HD.png]

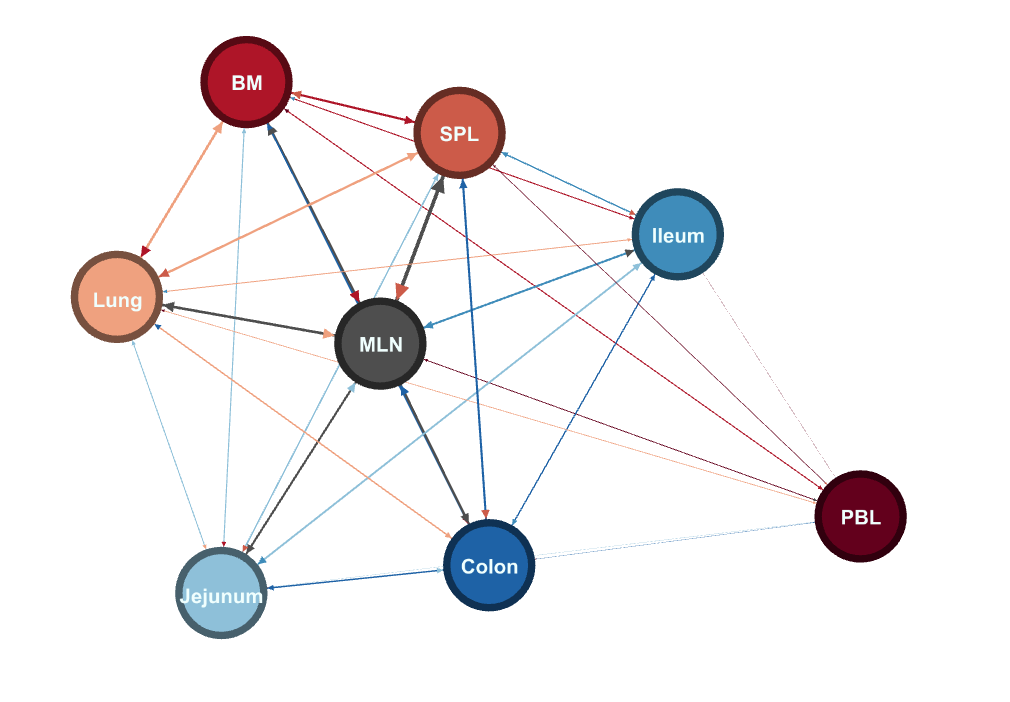

Supplement: Supplementary Materials File 5 — - by tissue distance heatmaps and networks: Heatmaps and Network representations as in Figure 1 one set of figure for each donor from (21). [file DataSheet_5.zip › Supplemental Materials file 5 -tissue distance heatmaps and networks/D145 by tissue graph.png]

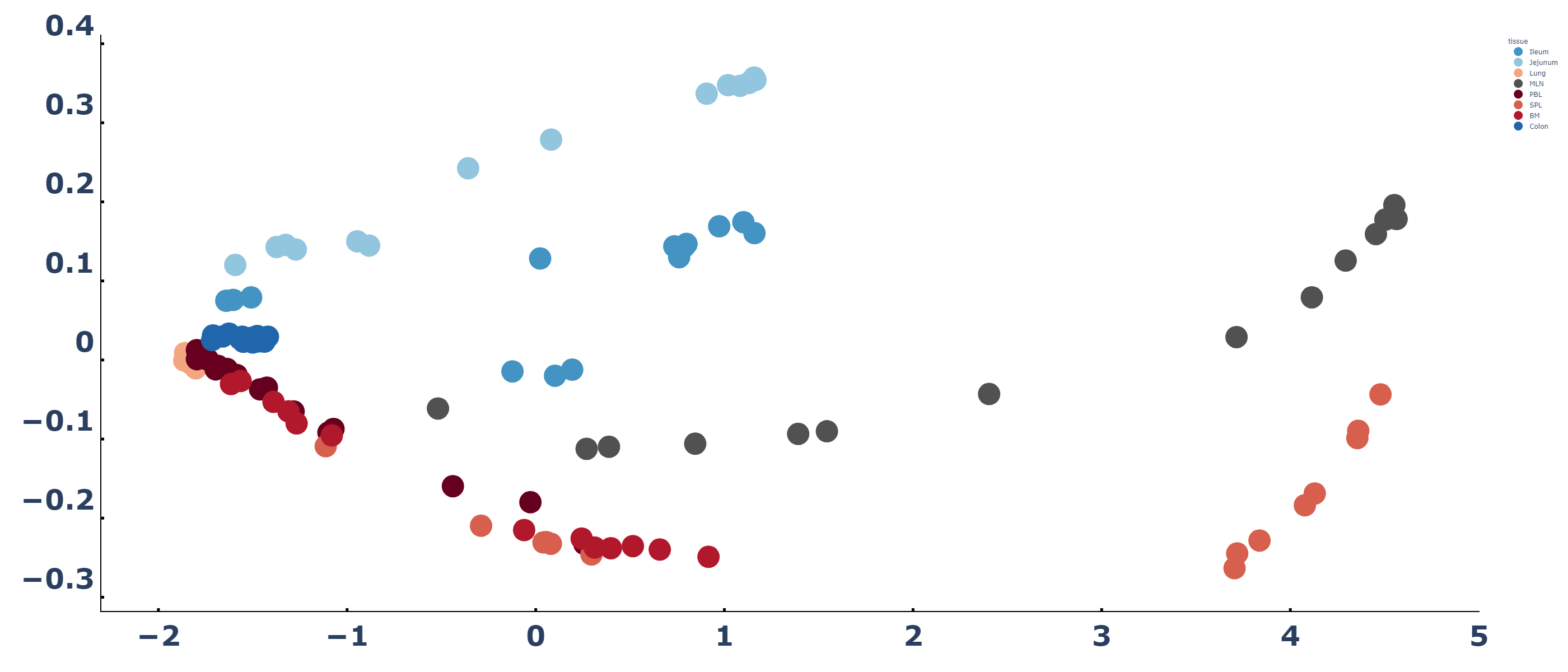

Supplement: Supplementary Materials File 7 — - figures of 2D PCA1_PCA2_PCA3 comparison based on sample distances: Relationships of the first three dimensions of the PCA for tissue sample signature differences calculated across each individual and color coded by tissue as in Figure 3 . One set of figures for each donor from (21). [file DataSheet_7.zip › Supplemental Materials file 7- figures of 2D PCA1_PCA2_PCA3 comparison based on sample distances/D181_PCA_xz_final.png]

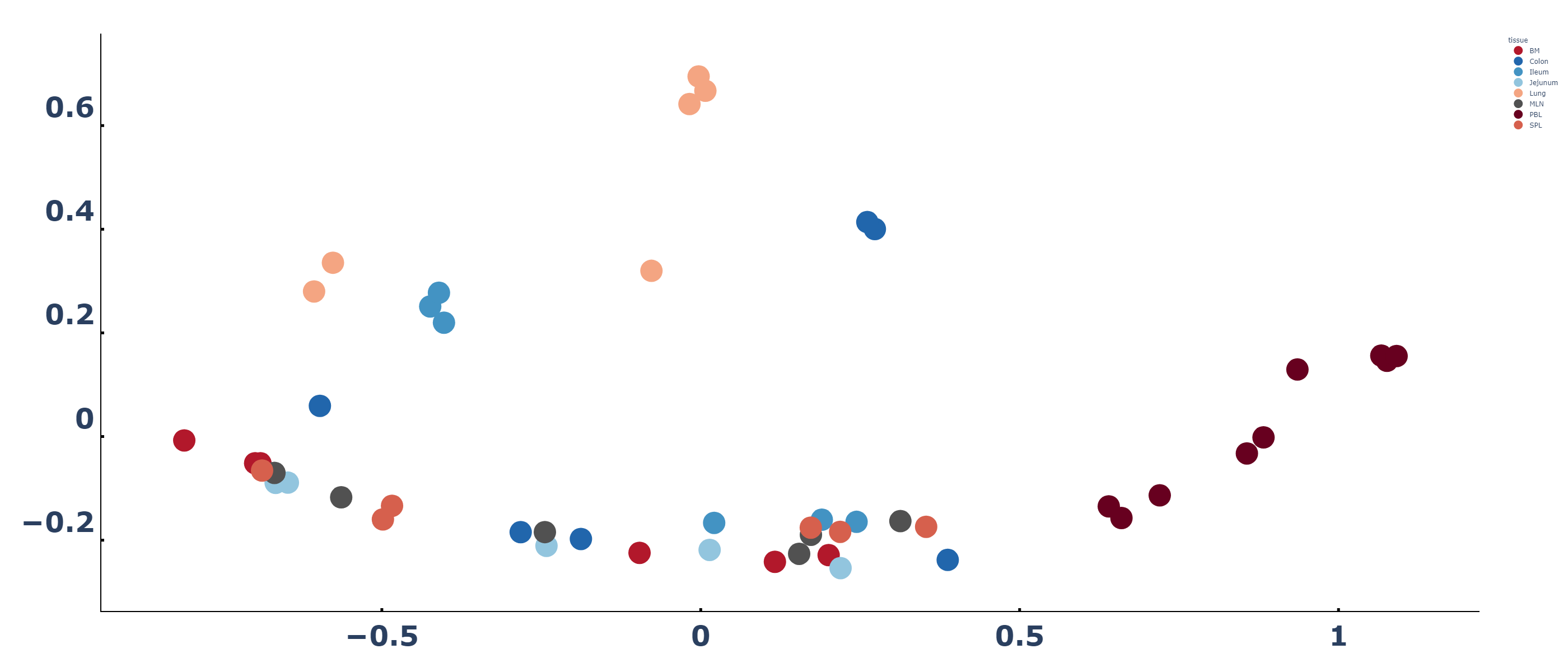

Supplement: Supplementary Materials File 7 — - figures of 2D PCA1_PCA2_PCA3 comparison based on sample distances: Relationships of the first three dimensions of the PCA for tissue sample signature differences calculated across each individual and color coded by tissue as in Figure 3 . One set of figures for each donor from (21). [file DataSheet_7.zip › Supplemental Materials file 7- figures of 2D PCA1_PCA2_PCA3 comparison based on sample distances/D182_PCA_xy_final.png]

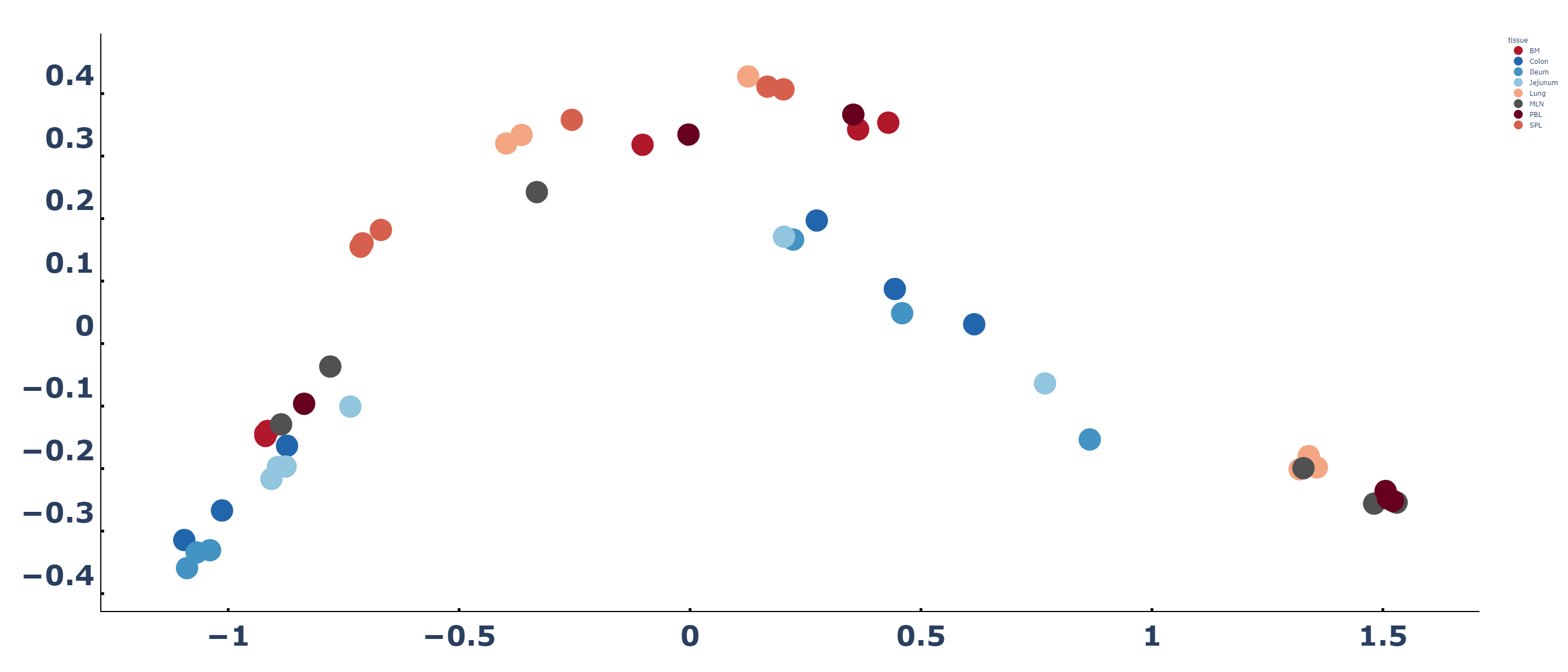

Supplement: Supplementary Materials File 7 — - figures of 2D PCA1_PCA2_PCA3 comparison based on sample distances: Relationships of the first three dimensions of the PCA for tissue sample signature differences calculated across each individual and color coded by tissue as in Figure 3 . One set of figures for each donor from (21). [file DataSheet_7.zip › Supplemental Materials file 7- figures of 2D PCA1_PCA2_PCA3 comparison based on sample distances/D145_PCA_xy_final.png]

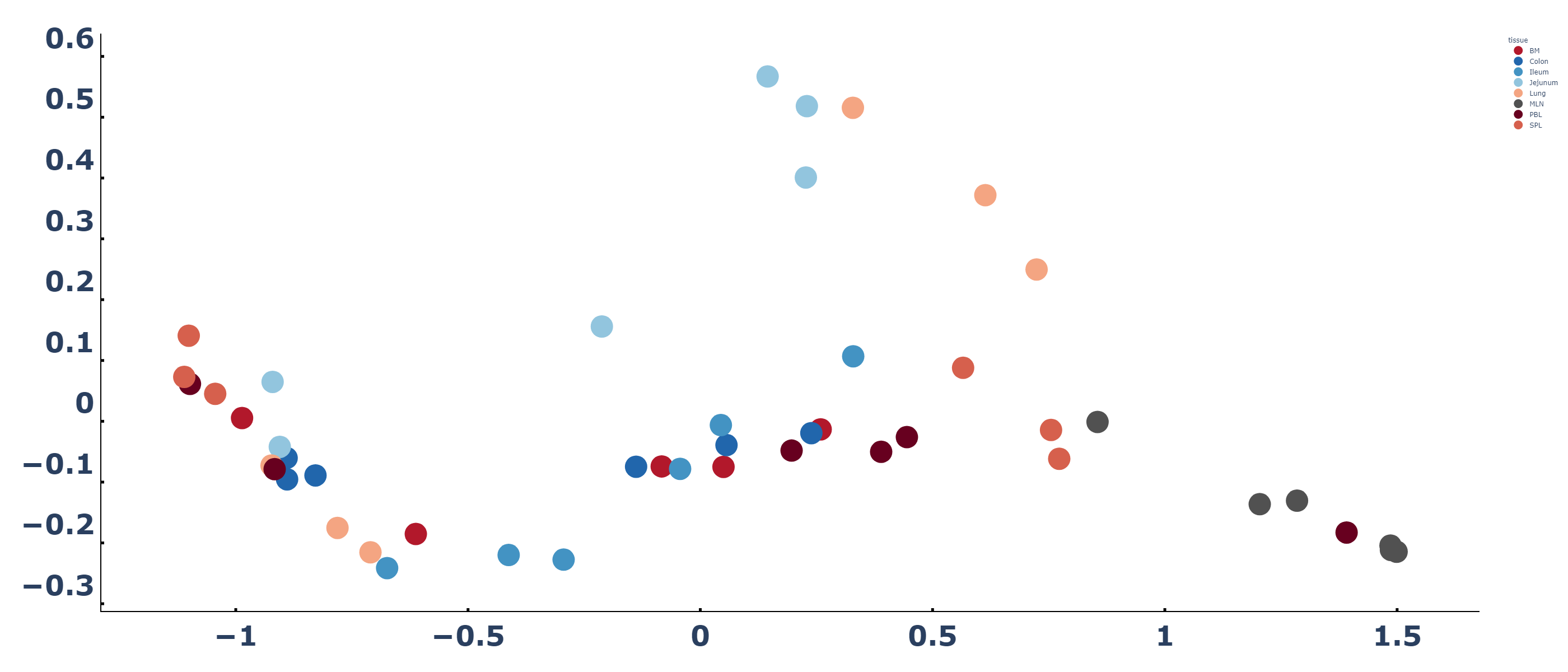

Supplement: Supplementary Materials File 7 — - figures of 2D PCA1_PCA2_PCA3 comparison based on sample distances: Relationships of the first three dimensions of the PCA for tissue sample signature differences calculated across each individual and color coded by tissue as in Figure 3 . One set of figures for each donor from (21). [file DataSheet_7.zip › Supplemental Materials file 7- figures of 2D PCA1_PCA2_PCA3 comparison based on sample distances/D168_PCA_xz_final.png]

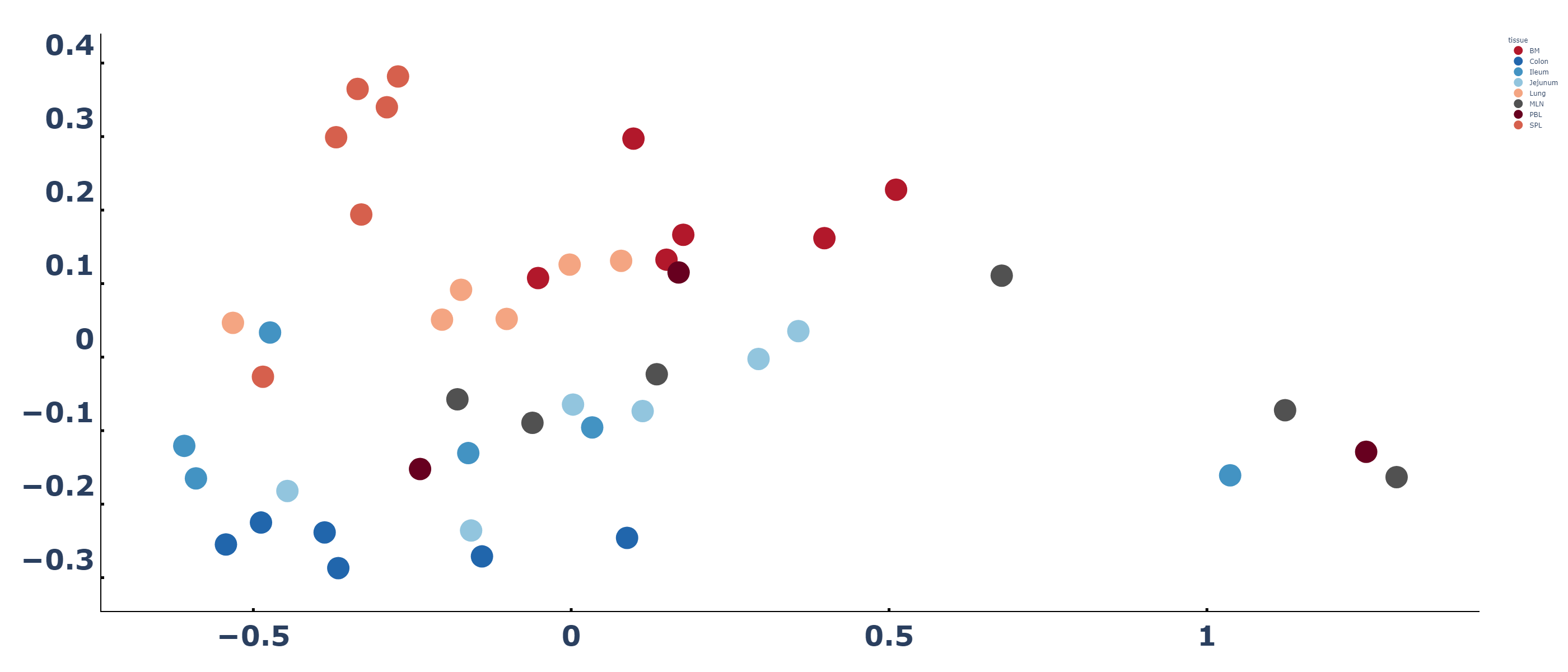

Supplement: Supplementary Materials File 7 — - figures of 2D PCA1_PCA2_PCA3 comparison based on sample distances: Relationships of the first three dimensions of the PCA for tissue sample signature differences calculated across each individual and color coded by tissue as in Figure 3 . One set of figures for each donor from (21). [file DataSheet_7.zip › Supplemental Materials file 7- figures of 2D PCA1_PCA2_PCA3 comparison based on sample distances/D149_PCA_xz_final.png]

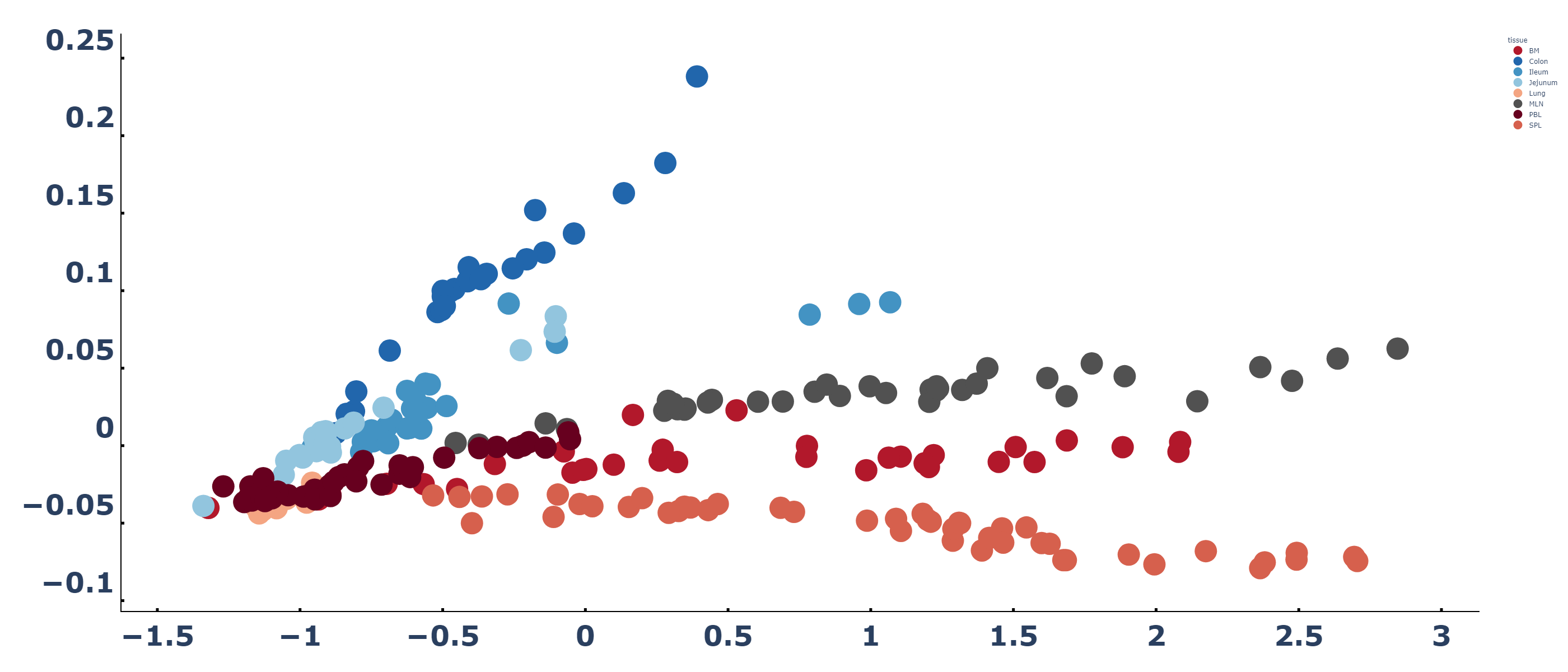

Supplement: Supplementary Materials File 7 — - figures of 2D PCA1_PCA2_PCA3 comparison based on sample distances: Relationships of the first three dimensions of the PCA for tissue sample signature differences calculated across each individual and color coded by tissue as in Figure 3 . One set of figures for each donor from (21). [file DataSheet_7.zip › Supplemental Materials file 7- figures of 2D PCA1_PCA2_PCA3 comparison based on sample distances/D207_PCA_xz_final.png]

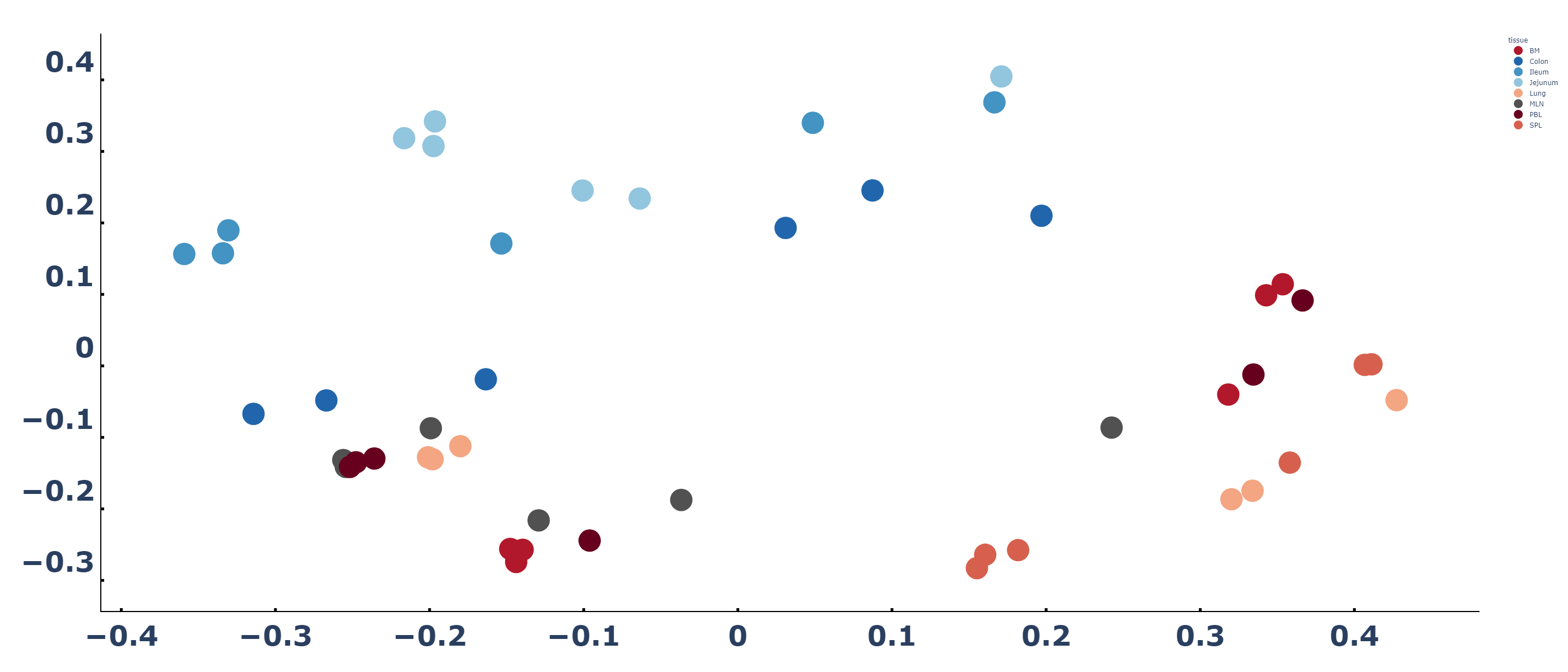

Supplement: Supplementary Materials File 7 — - figures of 2D PCA1_PCA2_PCA3 comparison based on sample distances: Relationships of the first three dimensions of the PCA for tissue sample signature differences calculated across each individual and color coded by tissue as in Figure 3 . One set of figures for each donor from (21). [file DataSheet_7.zip › Supplemental Materials file 7- figures of 2D PCA1_PCA2_PCA3 comparison based on sample distances/D145_PCA_yz_final.png]

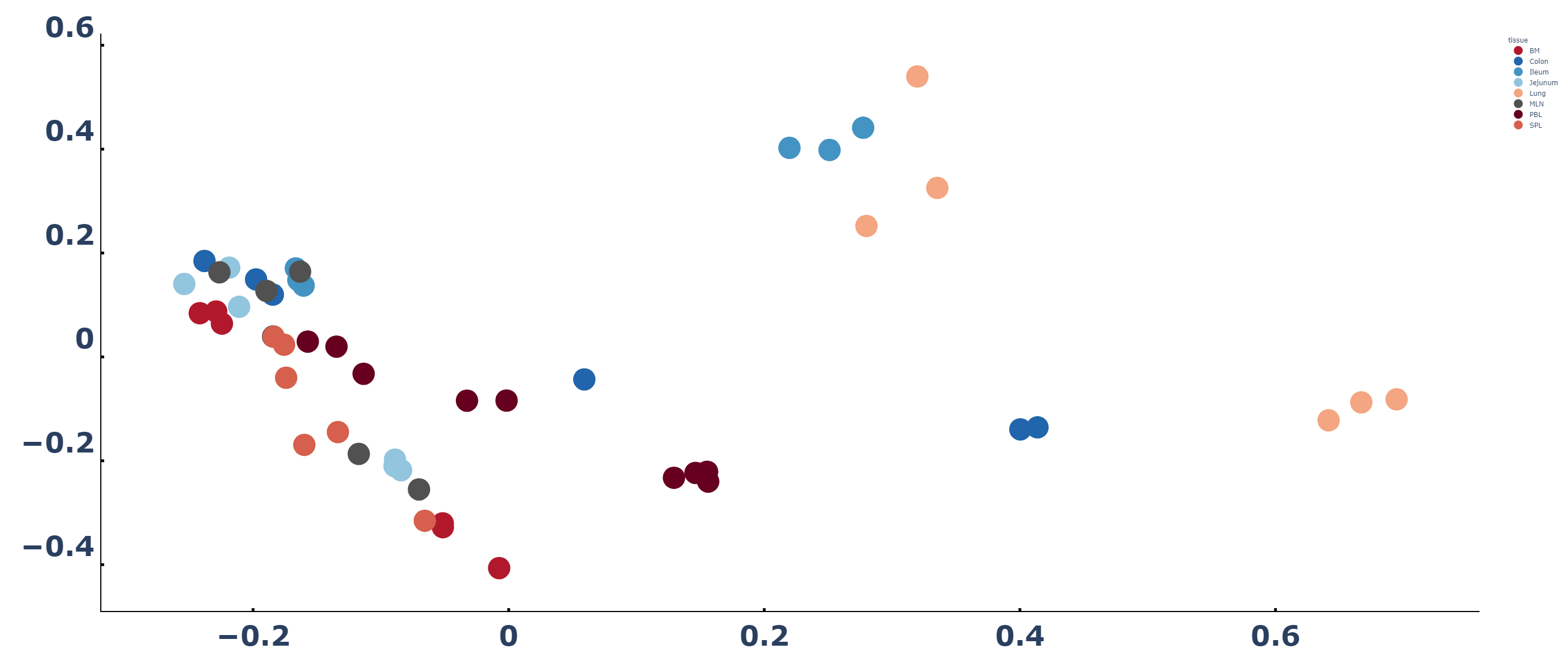

Supplement: Supplementary Materials File 7 — - figures of 2D PCA1_PCA2_PCA3 comparison based on sample distances: Relationships of the first three dimensions of the PCA for tissue sample signature differences calculated across each individual and color coded by tissue as in Figure 3 . One set of figures for each donor from (21). [file DataSheet_7.zip › Supplemental Materials file 7- figures of 2D PCA1_PCA2_PCA3 comparison based on sample distances/D182_PCA_yz_final.png]

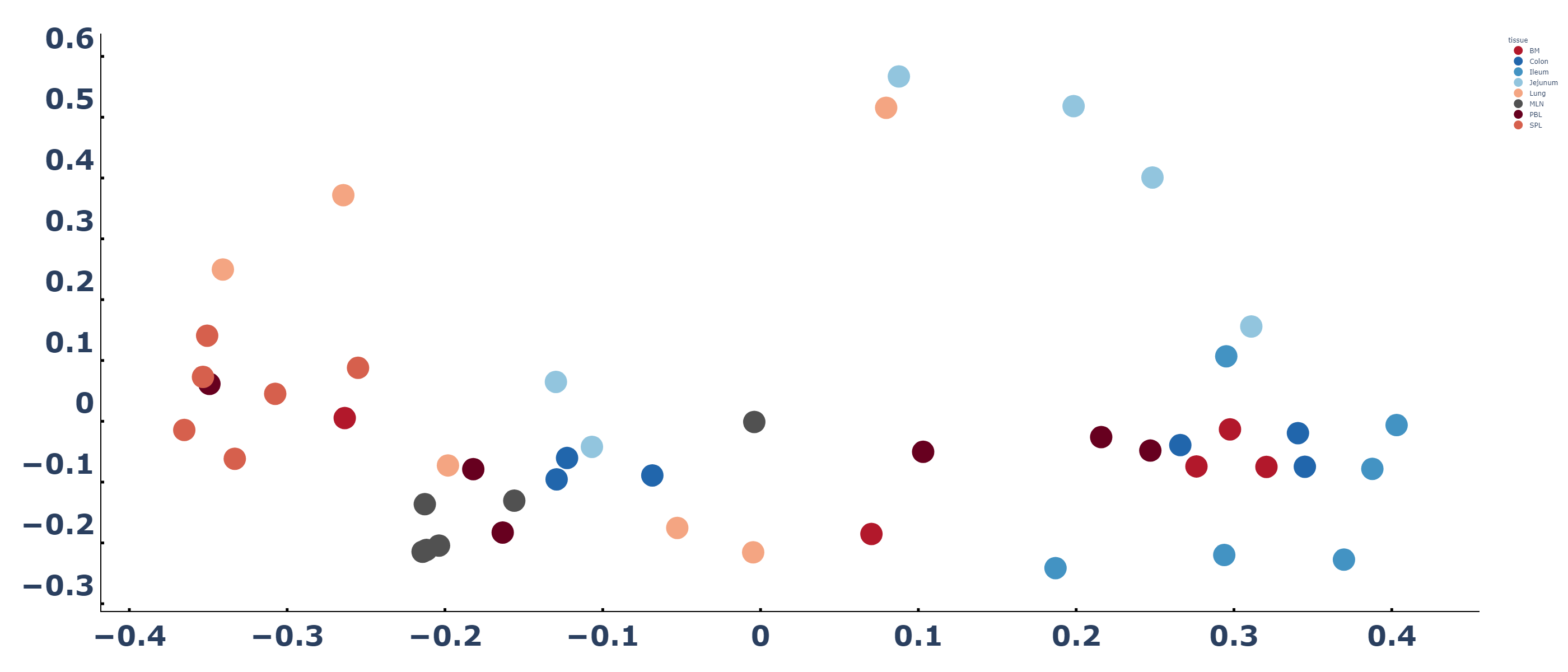

Supplement: Supplementary Materials File 7 — - figures of 2D PCA1_PCA2_PCA3 comparison based on sample distances: Relationships of the first three dimensions of the PCA for tissue sample signature differences calculated across each individual and color coded by tissue as in Figure 3 . One set of figures for each donor from (21). [file DataSheet_7.zip › Supplemental Materials file 7- figures of 2D PCA1_PCA2_PCA3 comparison based on sample distances/D168_PCA_yz_final.png]

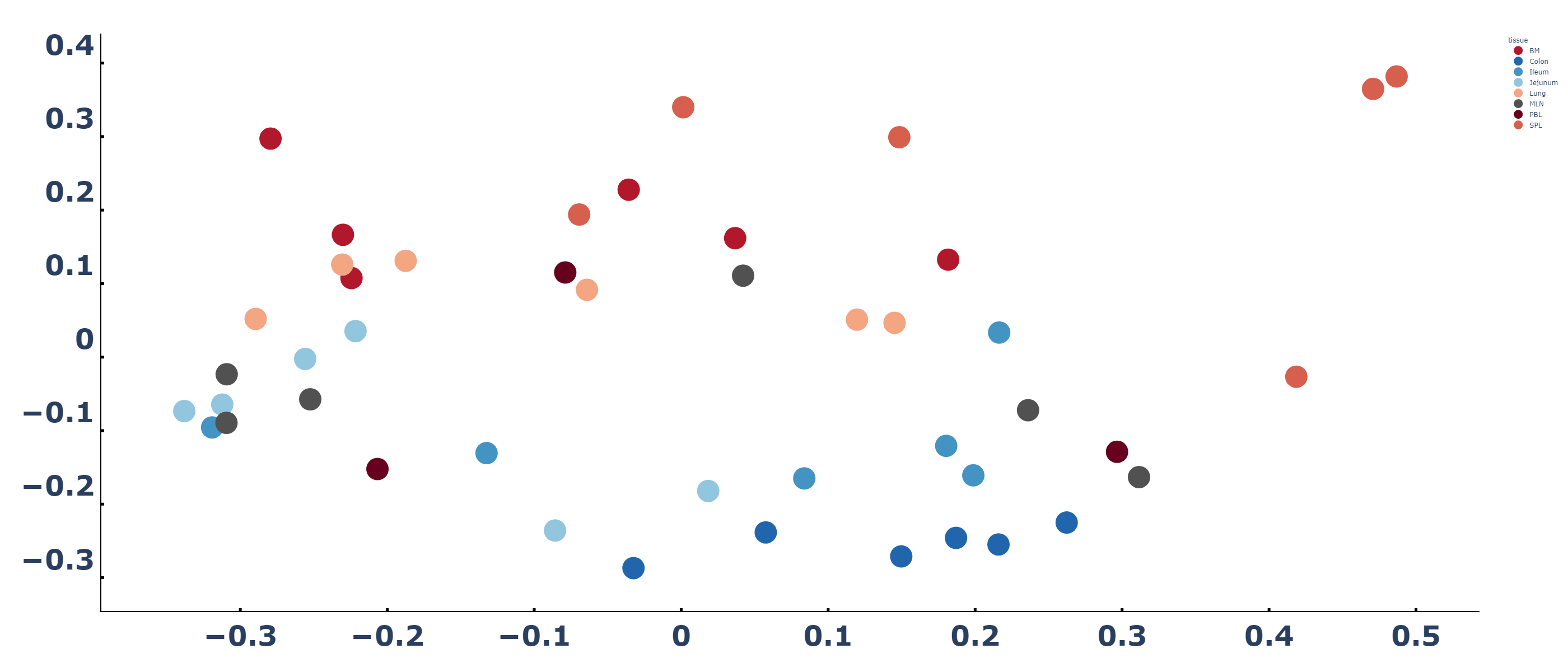

Supplement: Supplementary Materials File 7 — - figures of 2D PCA1_PCA2_PCA3 comparison based on sample distances: Relationships of the first three dimensions of the PCA for tissue sample signature differences calculated across each individual and color coded by tissue as in Figure 3 . One set of figures for each donor from (21). [file DataSheet_7.zip › Supplemental Materials file 7- figures of 2D PCA1_PCA2_PCA3 comparison based on sample distances/D149_PCA_yz_final.png]

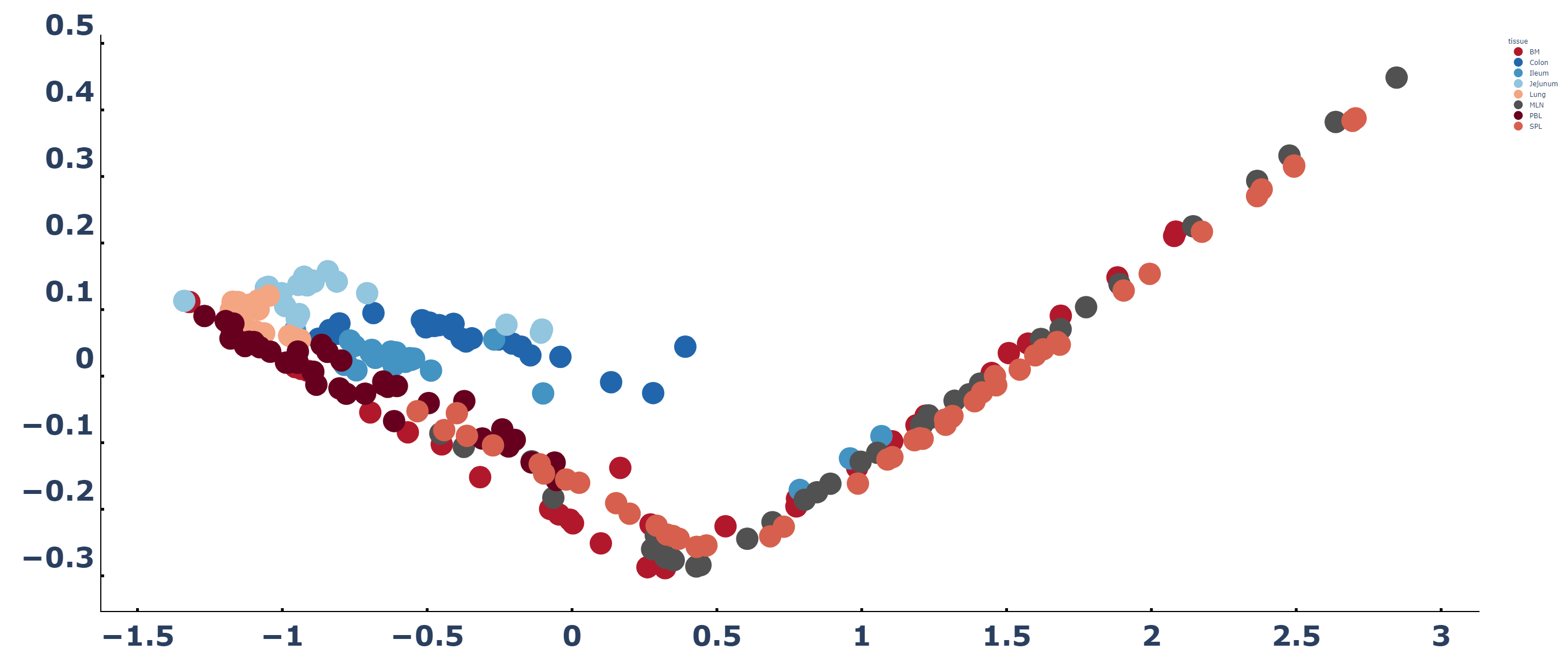

Supplement: Supplementary Materials File 7 — - figures of 2D PCA1_PCA2_PCA3 comparison based on sample distances: Relationships of the first three dimensions of the PCA for tissue sample signature differences calculated across each individual and color coded by tissue as in Figure 3 . One set of figures for each donor from (21). [file DataSheet_7.zip › Supplemental Materials file 7- figures of 2D PCA1_PCA2_PCA3 comparison based on sample distances/D207_PCA_xy_final.png]

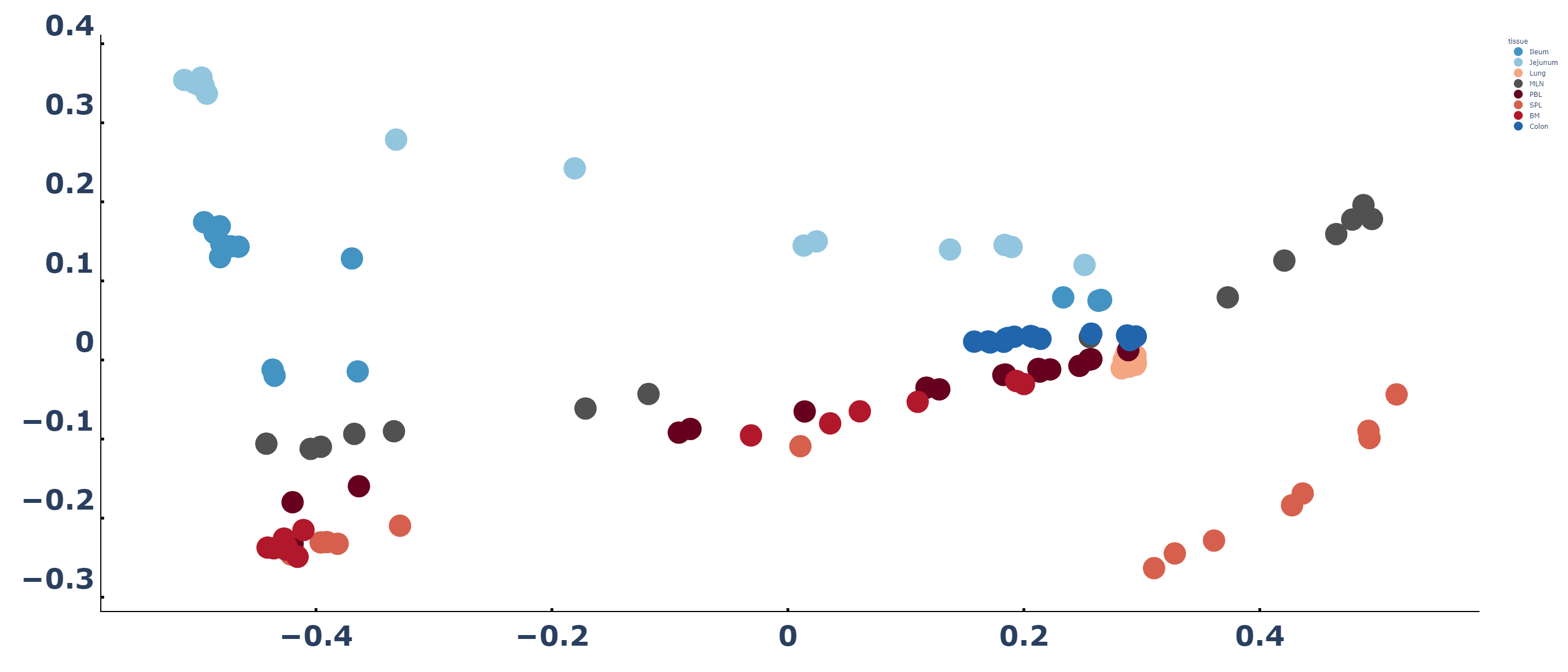

Supplement: Supplementary Materials File 7 — - figures of 2D PCA1_PCA2_PCA3 comparison based on sample distances: Relationships of the first three dimensions of the PCA for tissue sample signature differences calculated across each individual and color coded by tissue as in Figure 3 . One set of figures for each donor from (21). [file DataSheet_7.zip › Supplemental Materials file 7- figures of 2D PCA1_PCA2_PCA3 comparison based on sample distances/D181_PCA_yz_final.png]

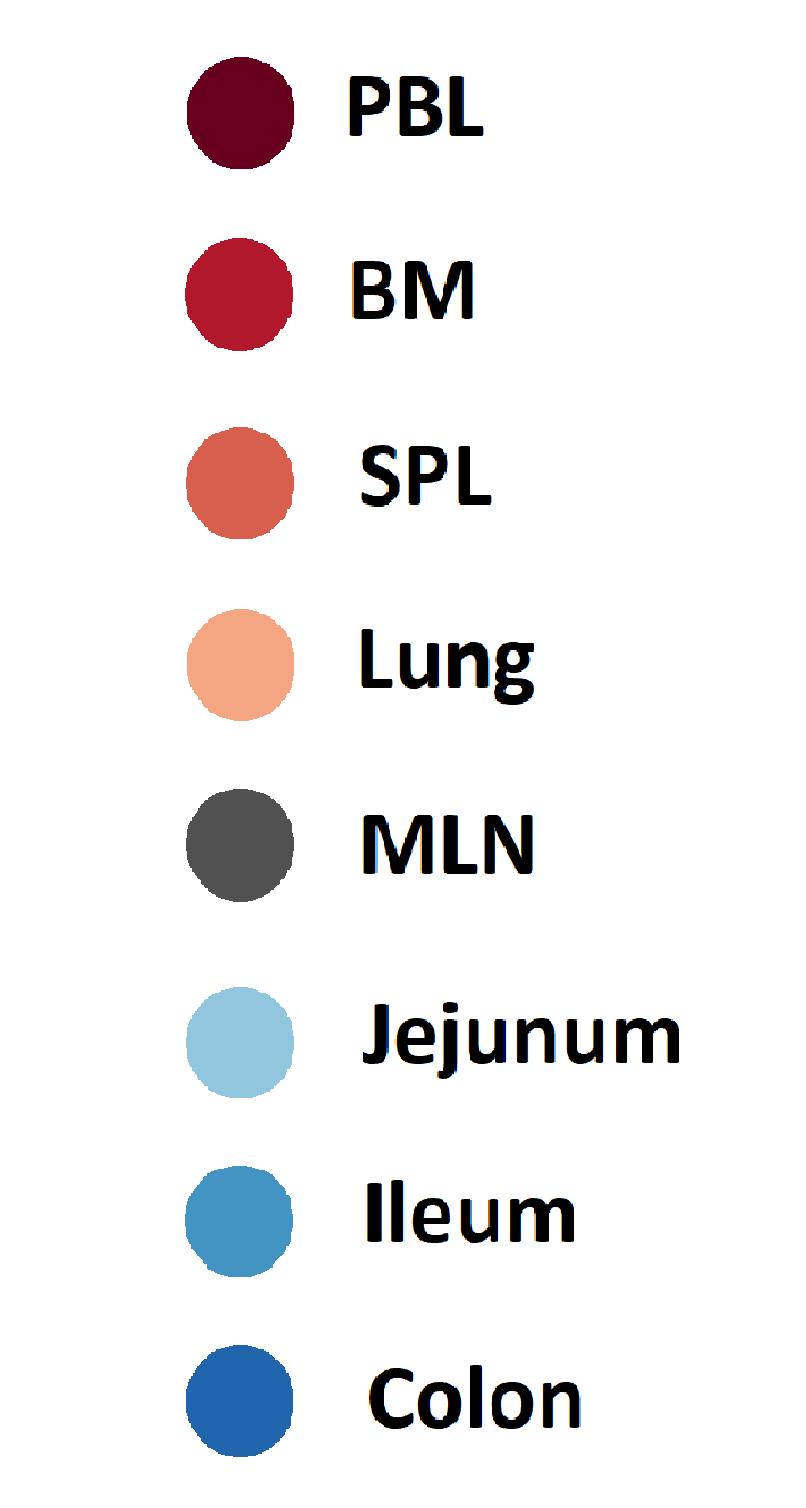

Supplement: Supplementary Materials File 7 — - figures of 2D PCA1_PCA2_PCA3 comparison based on sample distances: Relationships of the first three dimensions of the PCA for tissue sample signature differences calculated across each individual and color coded by tissue as in Figure 3 . One set of figures for each donor from (21). [file DataSheet_7.zip › Supplemental Materials file 7- figures of 2D PCA1_PCA2_PCA3 comparison based on sample distances/Legend final 2611.tif]

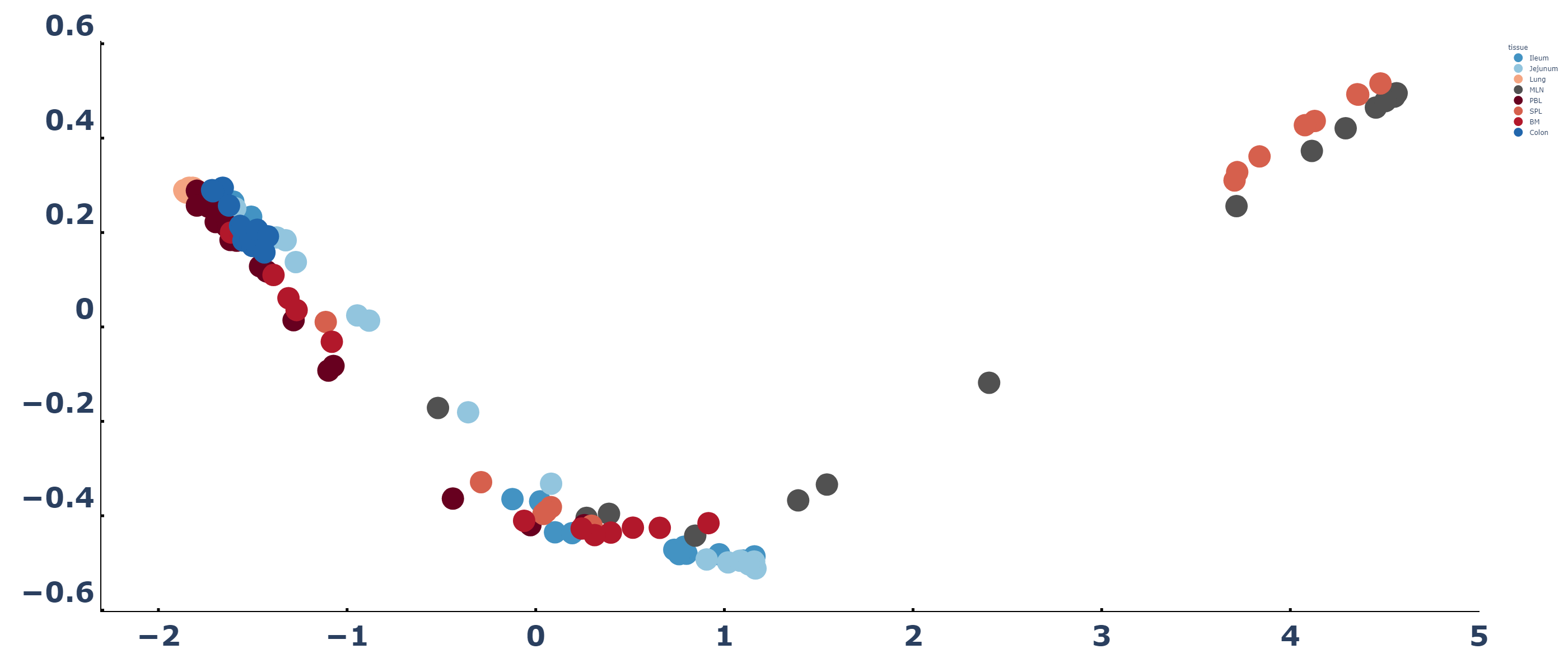

Supplement: Supplementary Materials File 7 — - figures of 2D PCA1_PCA2_PCA3 comparison based on sample distances: Relationships of the first three dimensions of the PCA for tissue sample signature differences calculated across each individual and color coded by tissue as in Figure 3 . One set of figures for each donor from (21). [file DataSheet_7.zip › Supplemental Materials file 7- figures of 2D PCA1_PCA2_PCA3 comparison based on sample distances/D181_PCA_xy_final.png]

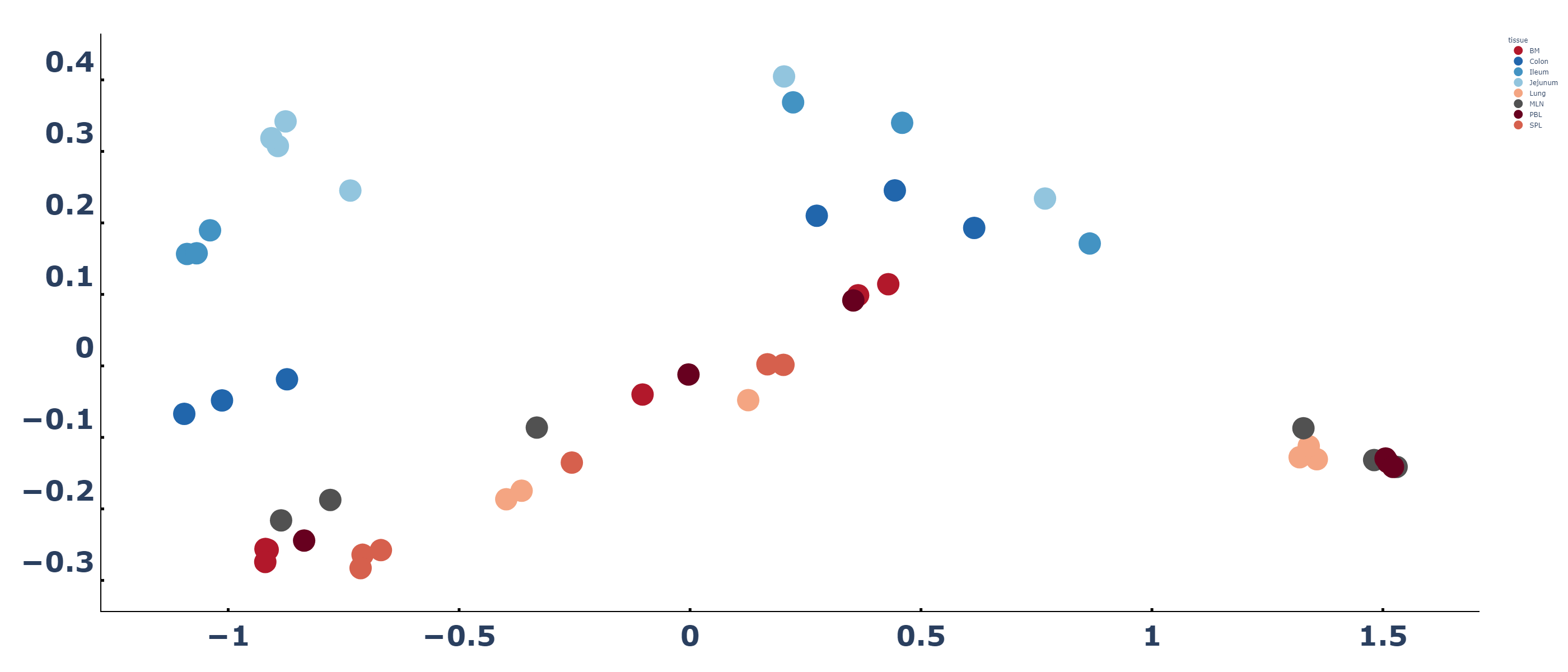

Supplement: Supplementary Materials File 7 — - figures of 2D PCA1_PCA2_PCA3 comparison based on sample distances: Relationships of the first three dimensions of the PCA for tissue sample signature differences calculated across each individual and color coded by tissue as in Figure 3 . One set of figures for each donor from (21). [file DataSheet_7.zip › Supplemental Materials file 7- figures of 2D PCA1_PCA2_PCA3 comparison based on sample distances/D145_PCA_xz_final.png]

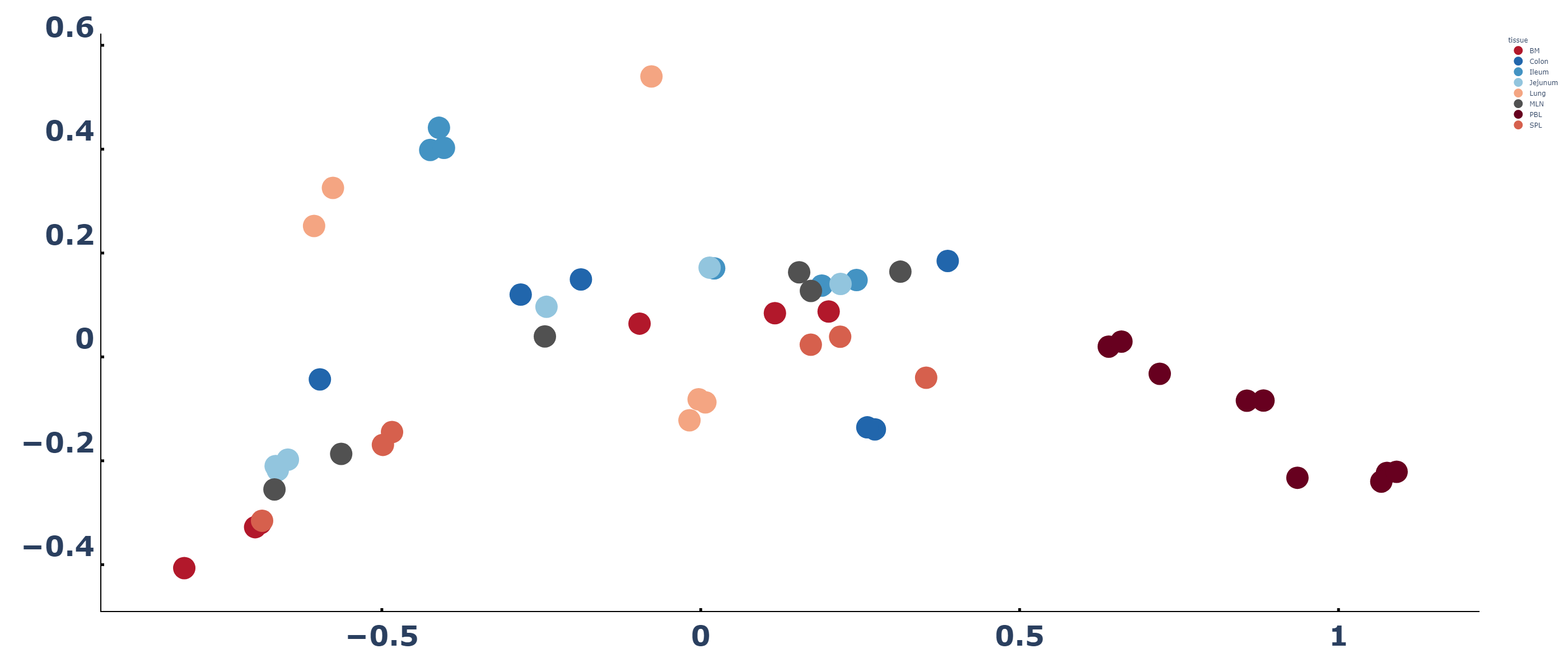

Supplement: Supplementary Materials File 7 — - figures of 2D PCA1_PCA2_PCA3 comparison based on sample distances: Relationships of the first three dimensions of the PCA for tissue sample signature differences calculated across each individual and color coded by tissue as in Figure 3 . One set of figures for each donor from (21). [file DataSheet_7.zip › Supplemental Materials file 7- figures of 2D PCA1_PCA2_PCA3 comparison based on sample distances/D182_PCA_xz_final.png]

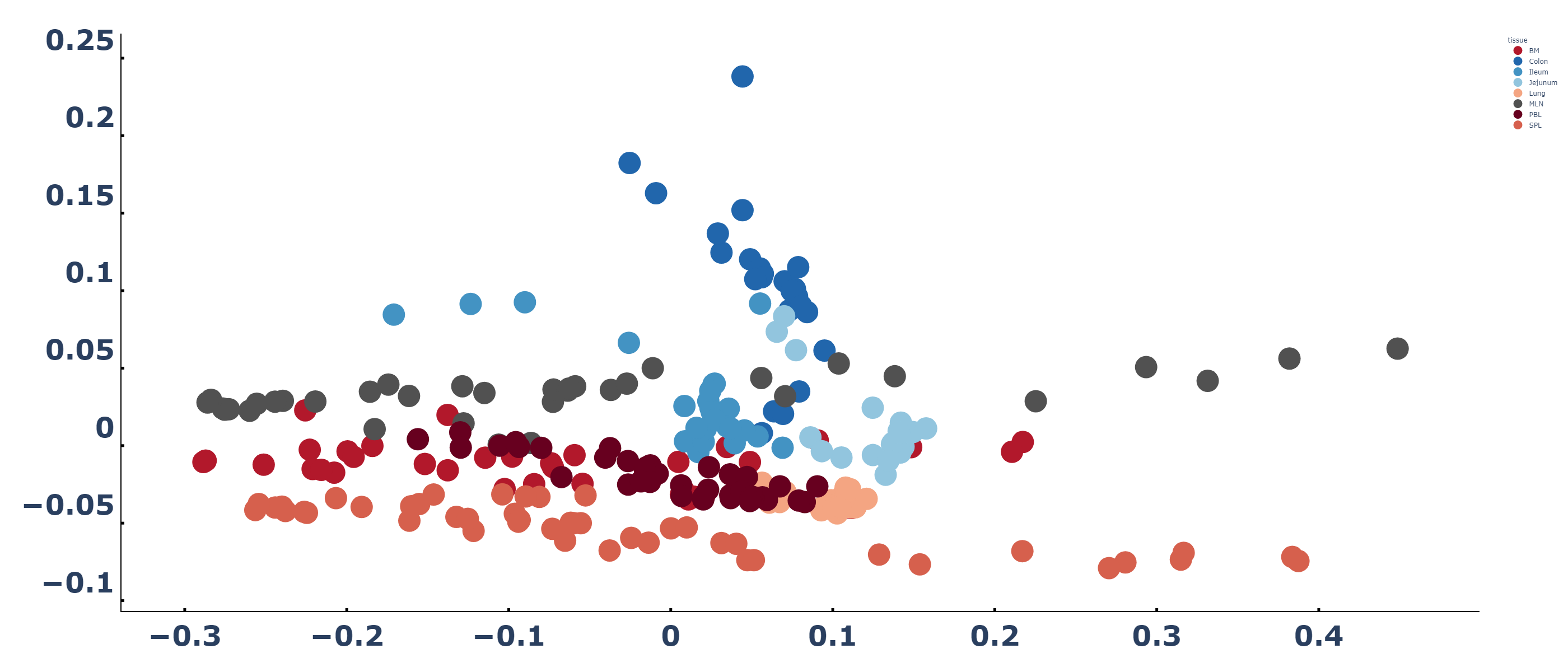

Supplement: Supplementary Materials File 7 — - figures of 2D PCA1_PCA2_PCA3 comparison based on sample distances: Relationships of the first three dimensions of the PCA for tissue sample signature differences calculated across each individual and color coded by tissue as in Figure 3 . One set of figures for each donor from (21). [file DataSheet_7.zip › Supplemental Materials file 7- figures of 2D PCA1_PCA2_PCA3 comparison based on sample distances/D207_PCA_yz_final.png]

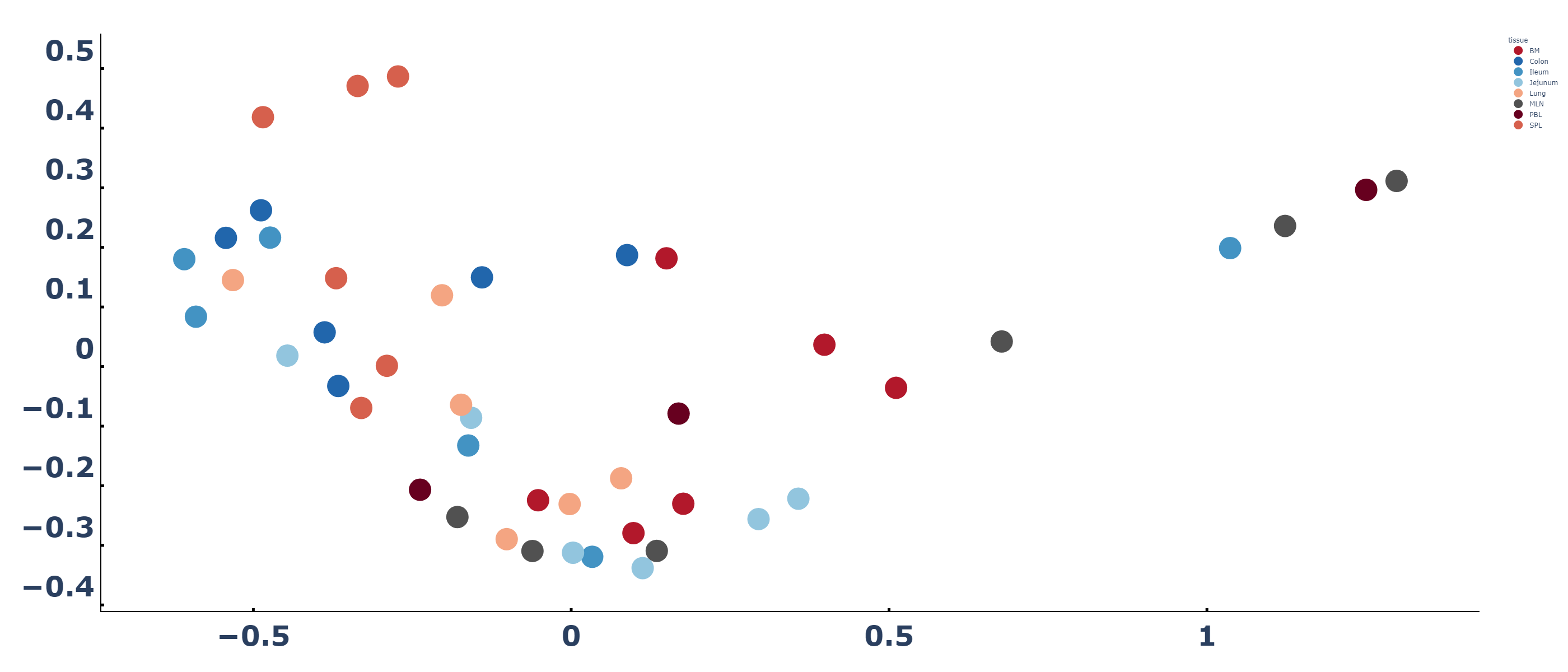

Supplement: Supplementary Materials File 7 — - figures of 2D PCA1_PCA2_PCA3 comparison based on sample distances: Relationships of the first three dimensions of the PCA for tissue sample signature differences calculated across each individual and color coded by tissue as in Figure 3 . One set of figures for each donor from (21). [file DataSheet_7.zip › Supplemental Materials file 7- figures of 2D PCA1_PCA2_PCA3 comparison based on sample distances/D149_PCA_xy_final.png]

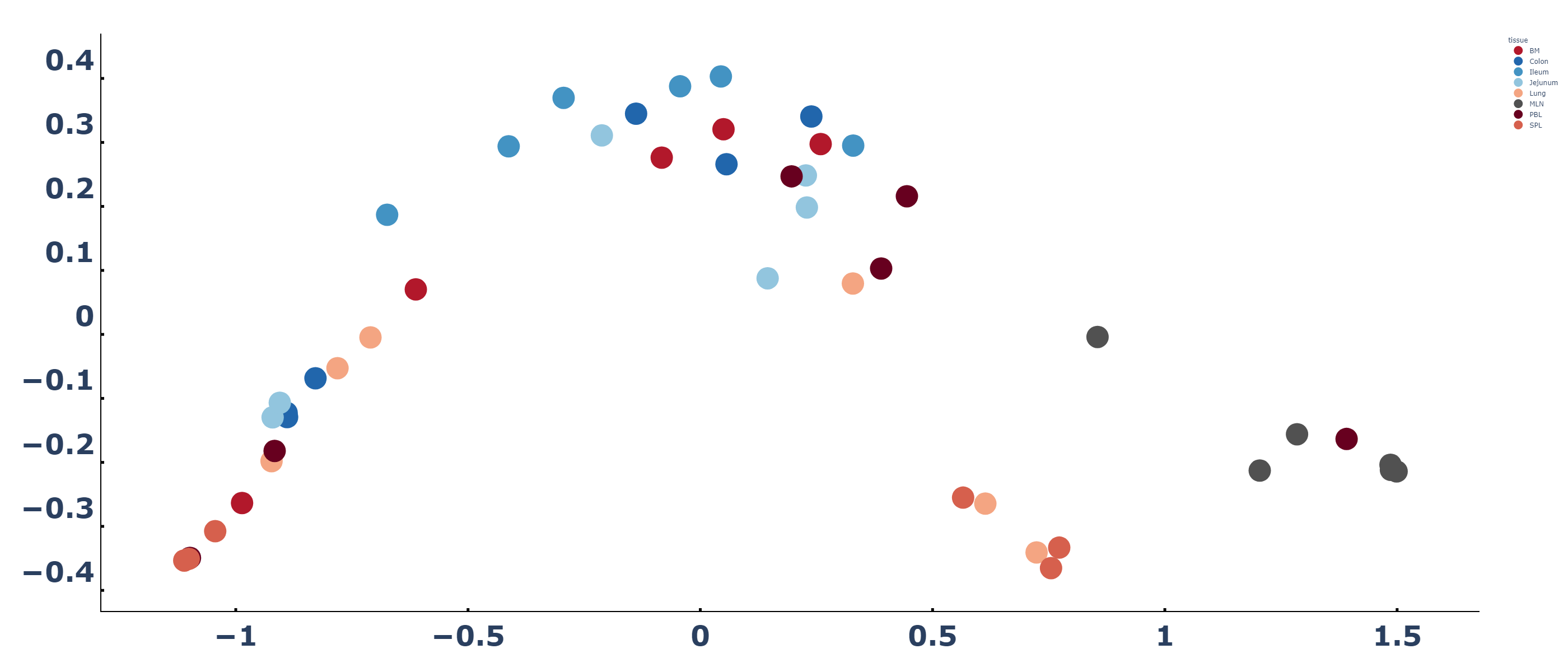

Supplement: Supplementary Materials File 7 — - figures of 2D PCA1_PCA2_PCA3 comparison based on sample distances: Relationships of the first three dimensions of the PCA for tissue sample signature differences calculated across each individual and color coded by tissue as in Figure 3 . One set of figures for each donor from (21). [file DataSheet_7.zip › Supplemental Materials file 7- figures of 2D PCA1_PCA2_PCA3 comparison based on sample distances/D168_PCA_xy_final.png]
