# Supplementary material for: Using Domain Based Latent Personal Analysis of B Cell Clone Diversity Patterns to Identify Novel Relationships Between the B Cell Clone Populations in Different Tissues
Source: Front Immunol. 2021 Apr 1;12:642673. doi: 10.3389/fimmu.2021.642673 (PMC8047331; doi:10.3389/fimmu.2021.642673)
Supplement: Supplementary file 10 [file Table_1.docx]

**Supplemental Table 1: Sample size of donor B cell repertoires from Meng et al. 2017^21^**

| **Donor** | **Library** | **Total copies** | **Unique sequences** | **Clones** |
| --- | --- | --- | --- | --- |
| D145 | 48 | 2,439,338 | 143,573 | 67,342 |
| D149 | 45 | 1,456,188 | 79,933 | 12,183 |
| D168 | 47 | 1,224,202 | 68,537 | 23,810 |
| D181 | 111 | 8,077,742 | 567,444 | 225,950 |
| D182 | 51 | 1,302,469 | 80,741 | 24,810 |
| D207 | 257 | 23,583,180 | 1,418,182 | 579,332 |

Library indicates the number of sequencing libraries generated per donor. Total copies refers to the total number of valid immunoglobulin VH region sequences. Unique sequences refers to the total number of unique in-frame sequences without a stop codon (productive rearrangements), that are found in at least two copies. Clones refers to the number of clonally related sequences, defined as having the same VH gene, the same CDR3 length and at least 85% sequence identity in the CDR3..
