# Supplementary material for: Using Domain Based Latent Personal Analysis of B Cell Clone Diversity Patterns to Identify Novel Relationships Between the B Cell Clone Populations in Different Tissues
Source: Front Immunol. 2021 Apr 1;12:642673. doi: 10.3389/fimmu.2021.642673 (PMC8047331; doi:10.3389/fimmu.2021.642673)
Supplement: Supplementary file 11 [file Table_2.docx]

**Supplemental Table 2: Variance described by first three dimensions (x,y,z) of sample entity distance PCA and distance from domain with signature length cutoff and without for donor B cell repertoires from Meng et al. 2017^21^**

1. Values of variance described signature lengths and cutoff when signature cutoff of ~ 0.5 is aimed for - as described in **Methods.**

| donor | x | y | z | total variance of xyz | sample signature length | average fraction of distance from domain |
| --- | --- | --- | --- | --- | --- | --- |
| D145 | 0.568 | 0.045 | 0.030 | 0.642 | 1000 | 0.500 |
| D149 | 0.307 | 0.073 | 0.045 | 0.426 | 100 | 0.510 |
| D168 | 0.489 | 0.049 | 0.031 | 0.569 | 200 | 0.600 |
| D182 | 0.286 | 0.062 | 0.046 | 0.394 | 500 | 0.543 |
| D181 | 0.909 | 0.027 | 0.027 | 0.963 | 9000 | 0.513 |
| D207 | 0.924 | 0.015 | 0.002 | 0.941 | 20000 | 0.510 |

1. Values when signature cutoff is extended to include most of the clones in the repertoire so as to enlarge the amount of variance described in the first three dimensions.

| donor | x | y | z | total variance of xyz | sample signature length | average fraction of distance from domain |  | average fraction of distance from domain |
| --- | --- | --- | --- | --- | --- | --- | --- | --- |
| D145 | 0.869 | 0.022 | 0.016 | 0.907 | 11000 | 0.81 |  |  |
| D149 | 0.789 | 0.023 | 0.018 | 0.830 | 1000 | 1 |  |  |
| D168 | 0.861 | 0.021 | 0.011 | 0.893 | 1900 | 0.8 |  |  |
| D182 | 0.843 | 0.027 | 0.018 | 0.888 | 2500 | 0.73 |  |  |
